# Comparative genomic analysis of nickel homeostasis in cable bacteria

**DOI:** 10.1186/s12864-024-10594-7

**Published:** 2024-07-15

**Authors:** Anwar Hiralal, Jeanine S. Geelhoed, Sinje Neukirchen, Filip J. R. Meysman

**Affiliations:** 1 Geobiology Research Group, University of Antwerp, Antwerp, Belgium; 2 Department of Biotechnology, Delft University of Technology, Delft, The Netherlands

**Keywords:** Cable bacteria, Nickel cofactor, Nickel homeostasis, RcnA, Genomics, *Candidatus* Electrothrix antwerpensis

## Abstract

**Background:**

Cable bacteria are filamentous members of the *Desulfobulbaceae* family that are capable of performing centimetre‑scale electron transport in marine and freshwater sediments. This long‑distance electron transport is mediated by a network of parallel conductive fibres embedded in the cell envelope. This fibre network efficiently transports electrical currents along the entire length of the centimetre‑long filament. Recent analyses show that these fibres consist of metalloproteins that harbour a novel nickel‑containing cofactor, which indicates that cable bacteria have evolved a unique form of biological electron transport. This nickel‑dependent conduction mechanism suggests that cable bacteria are strongly dependent on nickel as a biosynthetic resource. Here, we performed a comprehensive comparative genomic analysis of the genes linked to nickel homeostasis. We compared the genome‑encoded adaptation to nickel of cable bacteria to related members of the *Desulfobulbaceae* family and other members of the *Desulfobulbales* order.

**Results:**

Presently, four closed genomes are available for the monophyletic cable bacteria clade that consists of the genera *Candidatus* Electrothrix and *Candidatus* Electronema. To increase the phylogenomic coverage, we additionally generated two closed genomes of cable bacteria: *Candidatus* Electrothrix gigas strain HY10‑6 and *Candidatus* Electrothrix antwerpensis strain GW3‑4, which are the first closed genomes of their respective species. Nickel homeostasis genes were identified in a database of 38 cable bacteria genomes (including 6 closed genomes). Gene prevalence was compared to 19 genomes of related strains, residing within the *Desulfobulbales* order but outside of the cable bacteria clade, revealing several genome‑encoded adaptations to nickel homeostasis in cable bacteria. Phylogenetic analysis indicates that nickel importers, nickel‑binding enzymes and nickel chaperones of cable bacteria are affiliated to organisms outside the *Desulfobulbaceae* family, with several proteins showing affiliation to organisms outside of the *Desulfobacterota* phylum. Conspicuously, cable bacteria encode a unique periplasmic nickel export protein RcnA, which possesses a putative cytoplasmic histidine‑rich loop that has been largely expanded compared to RcnA homologs in other organisms.

**Conclusion:**

Cable bacteria genomes show a clear genetic adaptation for nickel utilization when compared to closely related genera. This fully aligns with the nickel‑dependent conduction mechanism that is uniquely found in cable bacteria.

**Supplementary Information:**

The online version contains supplementary material available at 10.1186/s12864-024-10594-7.

## Background

Cable bacteria are filamentous, multicellular bacteria that thrive in marine and freshwater sediments around the globe [1–3]. Cable bacteria possess a unique type of metabolism that depends on the ability to conduct electrons over centimetre‑scale distances [1, 4]. This long‑distance electron transport is mediated by conductive fibres that are embedded in the periplasm and run in parallel along the entire length of the filament [5, 6]. At the cell‑cell interfaces, the fibres are interconnected by a conductive cartwheel structure, which provides redundancy to the electrical network [7]. Together, the cartwheel‑connected fibre network forms the longest known electron transport channel in a single biological structure, and enables a unique division of metabolic labour between cells. The two redox half‑reactions of aerobic sulphide oxidation are carried out by different cells within the same filament that are up to centimetres apart but electrically connected [8, 9].

The cable bacterial fibres display extraordinary conductive properties, and achieve a high electrical conductivity not seen in other biological systems [6]. Recent investigations provided insight into the molecular structure that sustains these exceptional electrical properties. The protein fibres embed a nickel (Ni) cofactor new to biology, that consists of a nickel bis(dithiolene) (NiBiD) complex that forms an elongated, planar and highly conjugated structure [10, 11]. Multiple NiBiD cofactors are meticulously aligned along the longitudinal axis of the fibres, thus providing a one‑dimensional channel for efficient long‑distance electron transport [10]. The molecular structure and alignment of the NiBiD cofactor explains the organo‑metal like properties of the fibre network [12, 13] and demonstrates a manner via which macroscale supramolecular protein structures can attain exceptional conductivity.

The presence of Ni in the conductive fibres of cable bacteria is highly remarkable [10, 11]. Compared to other transition metals like iron (Fe) and copper (Cu), the incorporation of Ni into enzymes is rather limited, with only nine characterised enzymes known to have incorporated nickel in the active site [14, 15]. Moreover, all currently known metalloproteins involved in electron transport feature redox centres that include either Fe‑containing domains (e.g., haem groups, Fe‑S clusters) or Cu‑containing domains (e.g., cupredoxin), but never Ni‑containing cofactors [16].

Cable bacteria comprise two genera within the *Desulfobulbaceae* family: *Candidatus* Electrothrix, which is found in marine sediments, and *Candidatus* Electronema, which is mostly found in freshwater environments [17–20]. Other genera within the same family are *Desulfogranum*, *Desulfobulbus* and *Desulfolithobacter*, which, unlike cable bacteria, consist of unicellular sulphate reducers/sulphur disproportionators and neither have a periplasmic fibre network nor the capacity of long‑distance electron transport. Consequently, the acquisition of the NiBiD cofactor appears unique to cable bacteria, and therefore, it seems likely that this is reflected by genetic differences in Ni‑related genes between cable bacteria and other members of the *Desulfobulbaceae* family.

The periplasmic fibre network comprises a substantial fraction of the proteins in the periplasm [11]; it makes up for ~ 5% of the total biomass of the cable bacteria based on estimated volume and density. The periplasm has been shown to be specifically enriched in Ni, as a result of the incorporation of NiBiD cofactors [10]. As a consequence, cable bacteria require a suitable Ni supply to be able to synthesize the fibre network. Therefore, one expects this Ni dependency to be reflected by specific genomic adaptations for Ni homeostasis that are present in the cable bacteria but not the other members of the *Desulfobulbaceae* family.

Nickel homeostasis involves three major activities: (1) Ni import, (2) Ni binding by intracellular proteins, and (3) Ni export. As for Ni import, gram‑negative bacteria often take up Ni through the outer membrane via so‑called Ni‑siderophores [21], which are recognized by TonB‑dependent transporters (TBDTs), some of which are shown to be active for Ni [22–24]. Cytoplasmic uptake is achieved through two types of high‑affinity uptake systems: secondary permeases of the NiCoT family and ATP‑binding cassette (ABC) family proteins [25–27]. Intracellular Ni binding can reduce intracellular free Ni concentration and is achieved by several proteins: Ni‑binding enzymes, which utilize Ni for their enzymatic activity, and Ni‑binding chaperones, which are involved in the maturation of Ni‑binding enzymes and/or act as a Ni sequestrator [28]. Finally, Ni export can also reduce the intracellular free Ni concentration, and is attained by two types of transport: extracellular transport, in which intracellular Ni is exported to the extracellular environment, and periplasmic export, where Ni is moved from the cytoplasm to the periplasm [29, 30].

In this study, we aim to characterize the protein coding genes involved in nickel homeostasis in cable bacteria and compare these to other members of the *Desulfobulbaceae* family and *Desulfobulbales* order. To better cover the genetic diversity of cable bacteria, we generated two new complete genomes of members of the genus *Ca.* Electrothrix. We combine database searches with phylogenetic and gene synteny analysis to examine the genetic adaptation of nickel homeostasis in cable bacteria.

## Methods

### Creation of two new clonal enrichment cultures

Sediment was collected from a creek bed at Rattekaai salt marsh in the Netherlands (51.4391°N, 4.1697°E). Earlier studies at this location have confirmed the presence of cable bacteria in situ [2]. Sediment was sieved (< 1.1 mm) to remove large debris and fauna, homogenized and repacked in plastic core liners. A natural enrichment of cable bacteria was obtained after ~ 3 weeks by incubation of the sediment cores in oxygenated seawater, with a procedure as described previously [2].

To create clonal enrichment cultures of cable bacteria, we followed the same steps as described previously [31]. In brief, natural sediment from the site was autoclaved under N_2_ atmosphere, filled into cores and exposed to air. After a few days, when an oxic zone of ~ 1 mm had developed, a single cable bacterium filament was transferred to each core from a natural enrichment core. Cores were incubated at room temperature in the dark for several weeks. Two clonal enrichment cultures (HY10‑6 and GW3‑4) resulted from this inoculation procedure. To evaluate if a single cable bacterium strain was present in the enrichment cultures, we used V3‑V4 16S rRNA amplicon sequencing as described before [31].The two clonal cultures were subsequently maintained in the lab by regularly transferring a small amount of the clonal enrichment culture to freshly autoclaved sediment**.**

### Fluorescencein situhybridization, raman microscopy and atomic force microscopy

Individual filaments were collected from the fifth generation of the GW3‑4 clonal enrichment culture using small, custom‑developed glass hooks under a stereomicroscope [32]. Filaments were washed in MilliQ (mQ) water droplets (~ 20 µL) to remove sediment particles and salts. Fluorescence in situ hybridization (FISH) and Raman microscopy were performed following the same procedure as described in detail previously [31]. Raman spectra were collected using a Renishaw inVia™ Qontor® Confocal Raman microscope with a 532 nm excitation laser. To perform atomic force microscopy (AFM), individual cable bacteria filaments were transferred onto a 50 nm gold coated silicon wafer (Platypus technologies) and air dried. The wafer was attached to a 20 mm metal disc with a double sided carbon sticker. Imaging was performed with a XE‑100 atomic force microscope (Park Systems) equipped with an aluminium SPM probe with a tip radius < 10 nm (AppNano ACTA‑200) with nominal spring constant of 13–77 N/m. Topographic and amplitude data were recorded using tapping mode, and images were processed with Gwyddion software [33].

### Sediment sampling and DNA extraction

DNA was extracted from specific depth layers in the clonal enrichment cultures. To this end, individual sediment cores were sub‑sectioned at 0.3 cm depth resolution. DNA extraction was performed as previously described [20] using 0.25 g wet sediment as input. Briefly, DNA was purified from the wet sediment using enzymatic digestion (using RNase, Lysozyme and Proteinase K) followed by chloroform/isoamyl alcohol (24:1 v/v) extraction, subsequent precipitation with 10% polyethylene glycol, washing with 70% ethanol and finally dissolving in 10 mM Tris–HCl (pH 8) [20]. DNA was quantified using Qubit 3.0 and the Qubit dsDNA HS assay kit (Life Technologies, Thermo Fisher Scientific). To obtain sufficient DNA, the sections of 0.3‑0.6 cm and 0.6‑0.9 cm were pooled (total DNA quantity 4.2 μg for HY10‑6 and 5.3 μg for GW3‑4). Fragment length of extracted DNA was determined using a fragment analyser (Agilent 5300), showing an average length of 11 kbp for HY10‑6 and 12 kbp for GW3‑4.

### Single filament illumina sequencing

Individual filaments were retrieved from the HY10‑6 clonal enrichment culture using custom‑made glass hooks [32]. For the GW3‑4 sample, a small clump of filaments was collected, as insufficient DNA could be extracted from individual filaments in this sample. Cells were lysed at 95 °C for 15 min. DNA was amplified using multiple displacement amplification (MDA) with the REPLI‑g Single Cell Kit (Qiagen), according to the manufacturer’s instructions. Amplified DNA was used to perform Illumina HiSeq 2500 sequencing carried out by Eurofins Genomics, Konstanz, Germany.

### Metagenomic nanopore sequencing

DNA extracted from the sediment of the clonal enrichment cultures was sequenced using Nanopore MinION sequencing, carried out at the Neuromics support facility (Flanders Institute for Biotechnology, University of Antwerp). Nanopore library preparation was performed using the SQK‑LSK109 ligation sequencing kit (ONT), according to the manufacturer's instruction (DNA quantity 150 fmol). The EXP‑NBD114 native barcoding kit (ONT) was used to barcode and pool the samples at equimolar concentrations. The library was run on a FLO‑MIN106 MinION flowcell (9.4 chemistry, ONT) for 80 h, with half of the total library loaded initially and the other half added after 48 h.

### Basecalling and read processing

Raw Illumina data was quality‑checked using FastQC v0.11.750 (https://github.com/s‑andrews/FastQC) and MultiQC v1.751 [34]. To trim and filter reads, Trimmomatic v0.32 was used with the PE flag, a sliding window of 4, an average quality of 30 and a minimum length of 60 bp [35]. Raw Nanopore fast5 data were basecalled using Guppy v2.2.3 for MinION and the dna_r9.4.1_450bps_hac.cfg model from ONT. The data were explored using MinionQC v1.4.049 [36]. Qcat v1.0.1 (https://github.com/nanoporetech/qcat) was used to demultiplex and trim barcode and adapter sequences, using the following flags ‑b ‑k NBD103/NBD104 ‑‑trim ‑‑detect‑middle. Demultiplexed data amounted to 6.17 Gbp for GW3‑4 and 5.23 Gbp for HY10‑6. Reads were further processed using Filtlong v0.2.0 (https://github.com/rrwick/Filtlong), to remove reads < 4000 bp using ‑‑min_length 4000 and to remove low‑quality reads with < 80% base call accuracy using ‑‑min_mean_q 80. Porechop v0.2.3 (https://github.com/rrwick/Porechop) was used to check the reads for residual barcodes and adapters using default settings and the flag ‑‑min_split_read_size 4000.

### Genome assembly and polishing

The quality‑processed Nanopore data were used as input for Flye v1.2, using the ‑‑meta and ‑‑nano‑raw parameters [37, 38]. Contigs flagged to be circular by Flye were assessed to be truly circular by manual inspection of mapped reads that overlapped at the start and end of contigs in Tablet v1.21.02.08 [39]. Circular contigs belonging to the cable bacteria were identified using the genome classification option of GTDB‑Tk v2.3.2 [40]. The 16S rRNA gene sequence was retrieved using barrnap v0.9 (https://github.com/tseemann/barrnap).

Polishing as described previously [31] did not result in properly polished genomes for GW3‑4 and HY10‑6 (the 16S rRNA sequences changed in composition and length with every polishing step). Therefore, to polish the circular cable bacteria contigs, two rounds of polishing with the Illumina reads, acquired by single‑filament amplification, were performed using Pilon v1.24 [41]. This resulted in 100% identical 16S rRNA gene sequences compared to Illumina‑only assembled 16S sequences and Sanger sequences obtained using a nested‑PCR approach applied to several single filaments [42]. Polishing also resulted in strongly reduced gene fragmentation: the average protein sequence size increased from 238 to 316 amino acids for GW3‑4 and from 187 to 330 amino acids for HY10‑6. Samtools v1.958 and minimap2 v2.1657 were used as a dependency for polishing [43, 44].

### Cable bacteria genome dataset

Including the two closed genomes generated in this study, the initial dataset included a total of 51 publicly available genomes of cable bacteria, of which 45 were incomplete genomes [18, 19, 42, 45] and 6 closed genomes [19, 31, 46]. Genomes were downloaded from the NCBI genome database (accessed 21 august 2023) and evaluated for genome completeness using CheckM2 v1.02 [47]. Genomes with < 50% completeness or > 10% contamination were removed from the dataset, resulting in a final dataset of 38 genomes of medium quality or better according to MIMAG standards [47]. Within this dataset, 17 genomes were of high quality (HQ) (> 90% completeness and < 5% contamination), of which 10 were chosen as species representatives. As a reference and for comparison, 19 species representatives of the *Desulfobulbales* order were added to the dataset. Supplementary Table S1 provides an overview of the whole dataset (38 cable bacteria genomes, 19 *Desulfobulbales* reference genomes) including genome accession numbers.

GTDB‑Tk v2.3.2 was used with standard parameters to identify and align single-copy conserved protein sequences within the genome dataset, resulting in the identification and alignment of 118 single-copy marker genes [40]. IQtree v1.6.12 was used to infer a phylogenomic tree from the aligned protein sequences, with 1000 ultrafast bootstrap replicates and the automatic best-fit model finder option [48–50]. To infer species relatedness, 16S rRNA gene sequences were extracted from the genomes and used for pairwise comparisons. In addition, pyani v0.2.12 option ANIb was used to determine the average nucleotide identity of the cable bacteria genomes [51].

### Genome annotation

Genome annotation for the generated GW3-4 and HY10-6 strains was performed using the NCBI Prokaryotic Genome Annotation Pipeline (PGAP) v6.7 [52]. Nearly all other genomes in the dataset used the annotation as provided by NCBI (accessed 21st of August 2023) to avoid confusion with alternative annotation software. The exceptions are *Ca*. Electronema sp. SY1, *Ca*. Electrothrix arhusiensis HOU-hqMAG, *Ca*. Electrothrix gigas HOU-hqMAG and *Ca*. Electrothrix sp. SY2, where gene loci were identified using prodigal v2.6.3 and annotated using Prokka v1.14.1 [53, 54]. Details are provided in table S1.

### Ni homeostasis gene search in the cable bacteria dataset

Validated Ni homeostasis protein sequences from the literature were used as queries against the NCBI Reference Sequences (RefSeq) database (accessed 01 August 2023) using the blastp suite v2.14.1 [55]. From the top 100 hits, the sequences with ≥ 50% local identity and ≤ 10^−30^ *E*‑value threshold were retained, aligned using Clustal‑Omega v1.2.4 with default parameters [56], and used as input for hmmbuild v.3.1b2 using default parameters to create hmm profiles. The hmm profiles were used as input for HMMER v3.4 [57] to search the cable bacteria and representative *Desulfobulbales* dataset for Ni homeostasis gene homologs. Best hits were analysed for conserved domain architecture using InterProScan v 5.52_86.0 [58], conserved localization using SignalP v5.0 [59] conserved transmembrane helices using TMHMM v2.0 [60] and for conserved key residues by multiple sequence alignments using Clustal‑Omega v1.2.4 [56], all using default parameters. Catalytic [Ni‑Fe]‑hydrogenase subunits were classified using the online webtool HydDB [61].

### Phylogenetic analysis

To identify homologous proteins for phylogenetic analysis, the RefSeq database was used (accessed 01 September 2023). Identified nickel homeostasis proteins found in the closed genomes of cable bacteria were used as input (with the exception for Nik/Opp/Dpp homologs, where sequences from all cable bacteria genomes were used as here sequences were lacking) for the blastp suite v2.14.1 [55]. Blastp hits were filtered for the best hit per query sequence using a ≥ 25% local identity and a ≤ 10^−30^ *E*‑value threshold. Hits and query sequences were used for multiple sequence alignment with Clustal‑Omega v1.2.4 and the following parameters: ‑‑max‑guidetree‑iterations = 100 ‑‑max‑hmm‑iterations = 100 ‑‑output‑order = tree‑order [56]. In addition, protein sequences with predicted transmembrane helices were aligned with Praline, using default parameters [62]. In addition to the individual protein sequences, the concatenated sequences of NikQOM (in that order) and database hits were taken and aligned using Clustal‑Omega v1.2.4, with identical parameters as described above. As some organisms contained an NikMN fusion protein instead of a single NikM, the alignment was trimmed at the C-terminal side to remove the NikN sequence. To calculate all maximum‑likelihood phylogenies, IQtree v1.6.12 was used with the automatic best‑fit model finder and 1000 ultrafast bootstrap replicates [50]. All phylogenies were visualized with FigTree 1.4.4 (http://tree.bio.ed.ac.uk/software/figtree/).

## Results

### Two new closed cable bacteria genomes and identification of a novel cable bacterium species

To generate additional closed genomes of cable bacteria, we created two clonal enrichment cultures (denoted GW3‑4 and HY10‑6) and verified their clonality by V3‑V4 16S rRNA amplicon sequencing as described previously [31]. The clonal enrichment culture GW3‑4 showed a dominant ASV corresponding to cable bacteria, with a relative abundance of 2.1%. The clonal enrichment culture HY10‑6 showed a single ASV corresponding to cable bacteria, with a relative abundance of 7.7%. Sequencing provided 6.2 Gbp of Nanopore raw data and 3.3 Gbp of Illumina HiSeq raw data for the GW3‑4 sample, and 5.2 Gbp Nanopore and 4.1 Gbp Illumina HiSeq raw data for the HY10‑6 sample (Table S2, S3 & Fig. S1). Assembly and polishing resulted in two closed genomes, with a genome size of 4.7 Mbp for GW3‑4 and a considerably smaller genome size of 3.6 Mbp for HY10‑6. Both genomes have two 16S‑23S‑5S rRNA loci (Table S4).

**Fig. 1:**
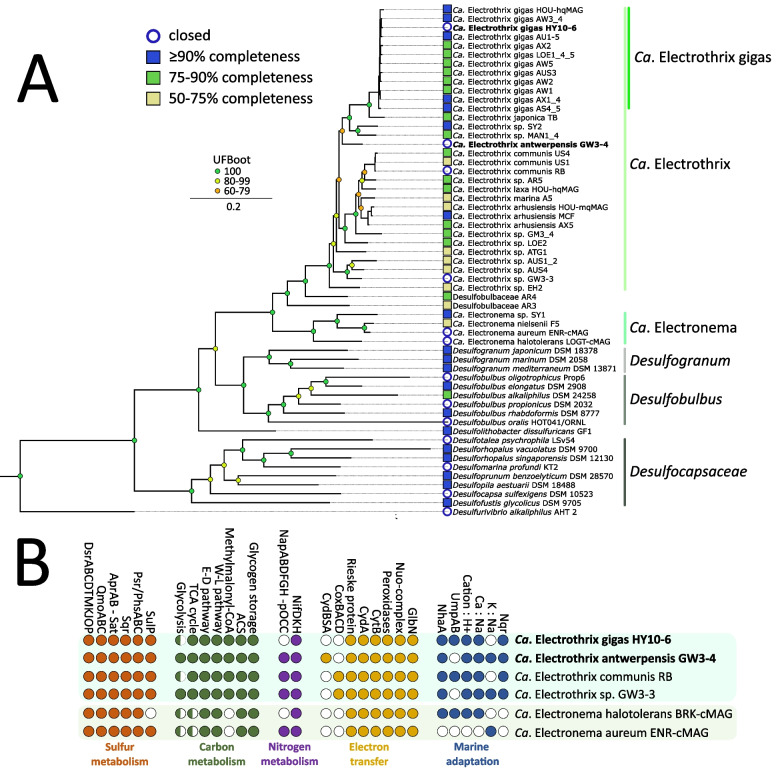
Genomic analyses of the new closed genomes of strain HY10-6 and strain GW3-4. **A** Maximum-likelihood phylogenomic tree of the cable bacteria and reference *Desulfobulbales* genomes used in this study. Phylogenomy inferred using IQtree according to the best-fit model (LG + F + R5). The HY10-6 and GW3-4 closed genomes are indicated in bold. **B** Metabolic potential of closed cable bacteria genomes. Complete pathways (filled circles) and pathways with at least one gene missing (half circles) are indicated. The HY10-6 and GW3-4 closed genomes are indicated in bold. For the full gene names and the respective locus tags, see Table S8

Based on whole genome average nucleotide identity (ANI) comparison, strain HY10‑6 belongs to the recently described *Ca.* Electrothrix gigas species [42]. Phylogenomic analysis placed strain GW3-4 within the candidate genus Electrothrix (Fig. 1A). ANI values of strain GW3-4 with the known *Ca*. Electrothrix species ranged from 77.4–82.3%, well below the established species delineation cut-off (95%) [63] (Table S5), and hence strain GW3‑4 represents a novel species of *Ca.* Electrothrix. Strain GW3‑4 grows as filaments and hybridizes with probe DSB706 targeting *Desulfobulbaceae* [64, 65], and detailed microscopy confirmed it is a cable bacterium (Fig. S2). Atomic force microscopy shows the pattern of parallel ridges on the outer surface, which are unique to cable bacteria. These ridges contain the periplasmic conductive fibres that extend over the cell–cell interfaces and thus are continuous along the entire filament length [4, 5] (Fig. S2A). Strain GW3-4 contains 23 (± 1) ridges, with cells ranging 0.4–0.5 µm in diameter and 3.4–6.3 µm in length (Figure S2A). Raman spectroscopy data collected with a green laser (532 nm) confirmed the presence of the unique spectrum of cable bacteria [10], with the two prominent frequency bands at 373 and 492 cm^−1^, which are indicative for the presence of the Ni‑containing NiBiD cofactor that is so‑far only found in cable bacteria [10, 11] (Fig. S2C). We propose the name *Ca*. Electrothrix antwerpensis for this new species.

The metabolic potential of *Ca*. E. antwerpensis GW3-4 is highly similar to that encoded in the closed genome of *Ca*. E. gigas HY10-6 and four other recently published closed genomes of cable bacteria (Fig. 1B) [19, 31, 46]. The potential is consistent with the metabolic model for cable bacteria suggested previously [18, 19, 31]. The gene repertoire for sulphur metabolism suggests that sulphur oxidation is based on a Dsr-Apr-Qmo-Sqr-Psr/Phs pathway, as was also suggested for sulphide oxidation in *Desulfurivibrio alkaliphilus* [66]. Genes encoding all subunits of the membrane-bound complex DsrMKJOP are present in the six closed genomes, showing the value of complete genomes compared to the first highly fragmented cable bacteria genomes in which *dsr(J)OP* were not detected [18, 31]. As in other *Ca*. Electrothrix species, carbon fixation potential exists for autotrophy via the Wood-Ljungdahl pathway and for heterotrophy via the methylmalonyl-CoA pathway and acetyl-CoA synthetase [9, 18, 67]. *Ca*. E. antwerpensis was cultivated with oxygen as electron acceptor. The genome encodes the same proteins hypothesized to be involved in electron transfer to oxygen that were detected in other cable bacteria: a Rieske Fe-S protein, a cytochrome bc complex subunit B, a homolog of cytochrome bd quinol oxidase subunit A and truncated hemoglobin [18]. Recently it has been shown that some cable bacteria genomes encode the membrane-bound cytochrome c oxidase complex CoxBACD (Fig. 1B) [19, 31]. In contrast, *Ca*. Electrothrix antwerpensis contains a gene cluster putatively encoding the three subunits of cytochrome bd quinol oxidase (CydBSA). The closest relatives of these subunits are found in the *Desulfobulbaceae*, e.g. ~ 72% sequence identity of CydA with *Desulfolithobacter dissulfuricans* and *Desulfobulbus propionicus*. In anaerobic sulphate reducers, cytochrome bd quinol oxidase plays a role in protection against oxygen [68]. In *Ca*. E. antwerpensis it could potentially couple oxygen reduction to proton translocation and hence energy conservation. The genome also contains an operon with *nap* genes and a gene encoding a putative periplasmic octaheme cytochrome c (pOCC), suggesting sulphide oxidation could also be coupled to dissimilatory nitrate reduction to ammonium (DNRA) [69].

The six closed cable bacteria genomes were combined with 32 publicly available incomplete genomes of cable bacteria (> 50% genome completeness, < 10% contamination; 38 cable bacteria genomes in total), as well as 19 reference genomes of the *Desulfobulbales* order for further analysis. Based on genome completeness and contiguity, 10 genomes were denoted as high‑quality species representatives in this study (Table S1). Phylogenomic analysis shows that the majority of major cable bacteria clades are covered with HQ MAGS (> 90% completeness, < 5% contamination) and/or closed genomes, and shows the genus *Desulfogranum* as the closest relative of the two cable bacteria genera *Candidatus* Electrothrix (further abbreviated as *Ca*. E.) and *Candidatus* Electronema (abbreviated as *Ca*. En.) (Fig. 1).

### Nickel import in cable bacteria

**Fig. 2:**
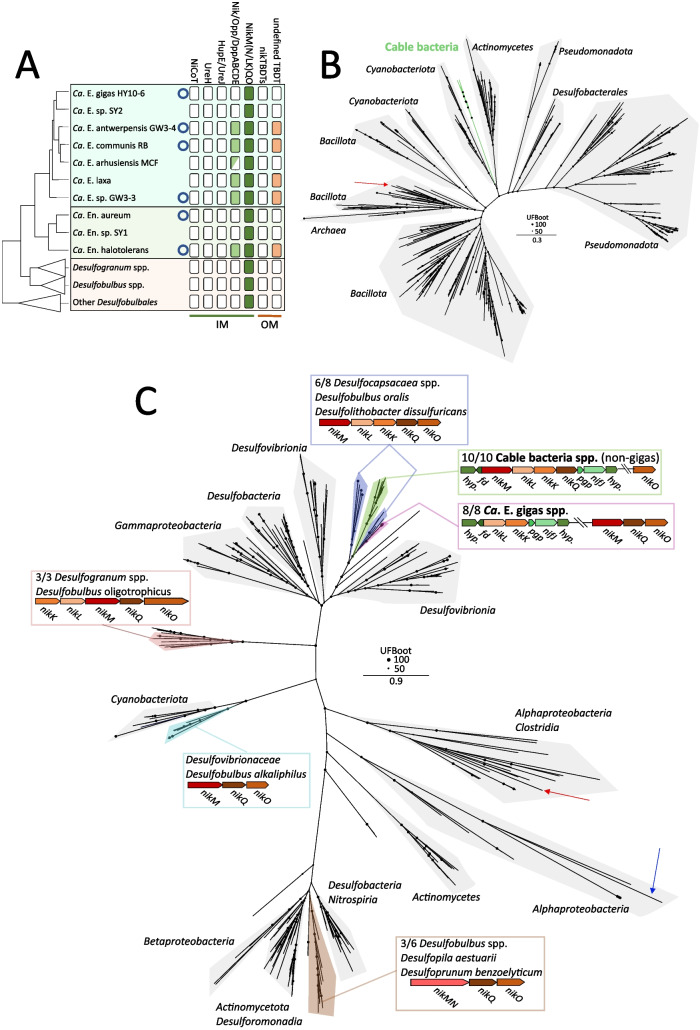
Presence and phylogeny of nickel import genes in cable bacteria. **A** Presence/absence table of nickel import genes in cable bacteria and related organisms of the *Desulfobulbales* order. For cable bacteria, only species representatives with HQ (no circle) or complete genomes (circle) are shown. For the *Desulfobulbales* species, presence is only indicated if the gene is found in two or more genomes. The presence of a full operon (filled) or partial operon (half‑filled) is indicated. **B** Maximum-likelihood phylogeny of cable bacteria Nik/Opp/DppA protein sequences and RefSeq similarity search hits. Phylogeny inferred using IQtree according to the best-fit model (model LG + R9). Black circles indicate ultrafast bootstrap values. Cable bacteria (green branches) form a monophyletic clade (UFBoot value = 70) between sequences from the phyla *Cyanobacteria* and *Actinomycetota*. The biochemically characterized Opp1A protein sequence of *S. aureus* is indicated with a red arrow. Only the lowest common shared taxonomy is indicated per clade. **C** Maximum likelihood phylogeny of concatenated NikMQO sequences of cable bacteria and refseq database hits. Phylogeny inferred using IQtree according to the best fit model (model LG4 + F + G4). Different *nikKLMNQO* operon structures (based on closed genomes) and their corresponding branches are indicated within the *Desulfobulbales* order. The NikMQO protein sequences of *Rhodobacter capsulatus* are indicated with the red arrow, and the CbiMQO sequences of *Rhodobacter capsulatus* are indicated with the blue arrow (root). Within the *Desulfogranum* cluster, the putative *nikO* homolog is doubled in length. Note that NikMQO sequences of *Ca.* E. gigas neither cluster with the other cable bacteria, nor share the same operon structure. The gene neighbourhood around the *nikKL* locus in *Ca.* E. gigas and the *nikMLKQ* locus in all other closed cable bacteria genomes is similar. *nifJ* = Pyruvate‑flavodoxin oxidoreductase, *pgp* = Phosphoglycolate phosphatase, *fd* = ferredoxin, *hyp*. = hypothetical protein

Active, high‑affinity Ni import across the cytoplasmic membrane in bacteria is known to be mediated by two mechanisms: secondary Ni/Co transporters and ABC‑binding cassette transporters. Secondary Ni/Co transporters include the NiCoT family of proteins (including NiCoT, NixA, HoxN, HupN, NicT and NhiF) which have been shown to be involved in Ni import in a variety of organisms [70–75] and the UreH and HupE/UreJ families of proteins, of which recombinant proteins of *R. palustris* and *C. necator* have been shown to confer Ni import [25]. Our genome analysis indicates that none of the specific secondary Ni/Co transporters are neither present in cable bacteria species representatives nor in the reference *Desulfobulbales* (Fig. 2A), nor in any of the other cable bacteria genomes (Table S6).

Of the ATP‑binding cassette (ABC) transporters, the NikABCDE transporter of *Escherichia coli* is the most well studied [76–80]. This transporter system is part of a large family of ABC transporters (Nik/Opp/Dpp family) that require ATP for active metal or peptide uptake and are found across many bacterial lineages [81, 82]. NikABCDE consists of a periplasmic binding protein (NikA), two transmembrane proteins located in the cytoplasmic membrane (NikBC) and two ATPases located in the cytoplasm (NikDE) [76, 83]. A subset of the cable bacteria genomes encodes homologs of the Nik/Opp/Dpp family importer. The putative Nik/Opp/Dpp family operon is fully present in the closed genomes of *Ca.* E. communis RB, *Ca.* E. sp. GW3‑3, *Ca.* E. antwerpensis and *Ca.* En. halotolerans, as well as in the single‑contig genome of *Ca.* E. laxa (Fig. 2A). Additionally, partial operons can be found in four MQ‑MAGs of the *Ca*. Electrothrix species (Table S6), which could be caused by genome incompleteness. The putative Nik/Opp/Dpp family operon is absent in *Ca.* E. gigas, for which a closed genome and seven HQ genomes are available (Fig. 2A, Table S6). Accordingly, this putative import system appears to be widely present, though not omnipresent in cable bacteria. Phylogenetic analysis of the Nik/Opp/DppA family substrate-binding subunit encoded by cable bacteria, together with the biochemically characterized NikA subunit of *Escherichia coli* and Opp1A subunit of *Staphylococcus aureus*, indicates that the subunit in cable bacteria is not an orthologue of *E. coli* NikA, but rather seems to be orthologous to *S. aureus* Opp1A (Fig. S3, Fig. 2B). Opp1A of *S. aureus* has been shown to promote nickel import [84], which could suggest a similar role in cable bacteria, although further biochemical characterization of the cable bacteria homolog is required. Similar genes are not found in other members of the order *Desulfobulbales* [85] (Fig. 2A). Phylogenetic analysis with hits from the RefSeq database similarity search shows that putative cable bacteria Nik/Opp/DppA protein sequences form a separate monophyletic sister clade to sequences of the *Cyanobacteria* and *Actinomycetota* phyla (Fig. 2B).

A second, less well characterized Ni import system that belongs to the energy‑coupling class of ABC transporters is the NikMNQO/NikM(K)LQO system, which was discovered by comparative genomics [81]. In these systems, NikO is predicted to be the sole ATPase, while all other components are predicted to be integrated into the cytoplasmic membrane, with no extra‑cytoplasmic subunit like NikA predicted to be present. The NikMNQO system is present across many bacterial lineages, while the NikM(K)LQO system is mostly found in *Pseudomonadota* and *Cyanobacterota* [81]. In our dataset, a putative NikM(K)LQO system is found in all closed and HQ cable bacteria genomes, whereas both NikMNQO and NikM(K)LQO systems are putatively found in all members of the *Desulfobulbales* order (Fig. 2A, Table S6). All cable bacteria NikMQO clusters are phylogenetically distantly related to NikMQO homologs of members of the *Desulfogranum* and *Desulfobulbus* genera (Fig. 2C). Additionally, the operon structure found in the genera *Desulfogranum* and *Desulfobulbus* differs to those found in cable bacteria (Fig. 2C). Instead, for cable bacteria, the gene operon structure of NikMLKQO found in the majority of closed genomes, except for *Ca.* E. gigas, is similar to the ones found in several *Desulfocapsaceae* species (Fig. 2C). Phylogenetic analysis of individual subunits indicates that the NikM, NikQ and NikO subunits of cable bacteria cluster contradistinctive to their phylogenomic relationship: individual NikMQO subunits of *Ca.* E. gigas species cluster separately from other *Ca*. Electrothrix species and are more closely related to sequences found in *Ca*. Electronema species and *Desulfolithobacter dissulfuricans* (Figs. 1, S3, S4, S5). Conversely, phylogenetic analyses of NikK and NikL homologs indicate a phylogenetic relationship between *Ca*. E. gigas and other *Ca*. Electrothrix species that concurs with their phylogenomic relationship (Figs. 1, S6, S7). To add to this, gene synteny analysis shows that the gene neighbourhood around the NikKL operon in *Ca.* E. gigas is similar to the NikMLKQO operon found in other closed genomes of cable bacteria (Fig. 2C), thus suggesting a potential gene rearrangement of this locus in *Ca*. E. gigas.

Active metal import across the outer membrane in gram‑negative bacteria is mediated through TonB‑dependent transporters (TBDTs) [21, 24]. A number of TBDTs are known to function as Ni(II) transporters: these include FecA3, FrpB4, CntO, Bll6948, Daro_3944 and Rgel01002199 and NikH [23, 81, 86–89]. Our analysis indicates that cable bacteria possess genes coding for several copies of TBDTs per genome, but none of these TBDTs are related to the known Ni(II) TBDTs (Fig. 2A, Table S6). However, cable bacteria that encode the putative Nik/Opp/Dpp family system contain a TBDT directly upstream of the corresponding operon. This undefined TBDT is not found in any other member of the *Desulfobulbales* order (Fig. 2A). Phylogenetic analysis shows that genes of this TBDT in cable bacteria form a monophyletic clade closely related to sequences of the *Chromatiaceae* family (class *Gammaproteobacteria*; Fig. S8). Biochemical characterization of these TBDTs is required to ascertain whether cable bacteria possess the capacity for active nickel uptake across the outer membrane.

### Intracellular nickel‑binding proteins in cable bacteria

**Fig. 3:**
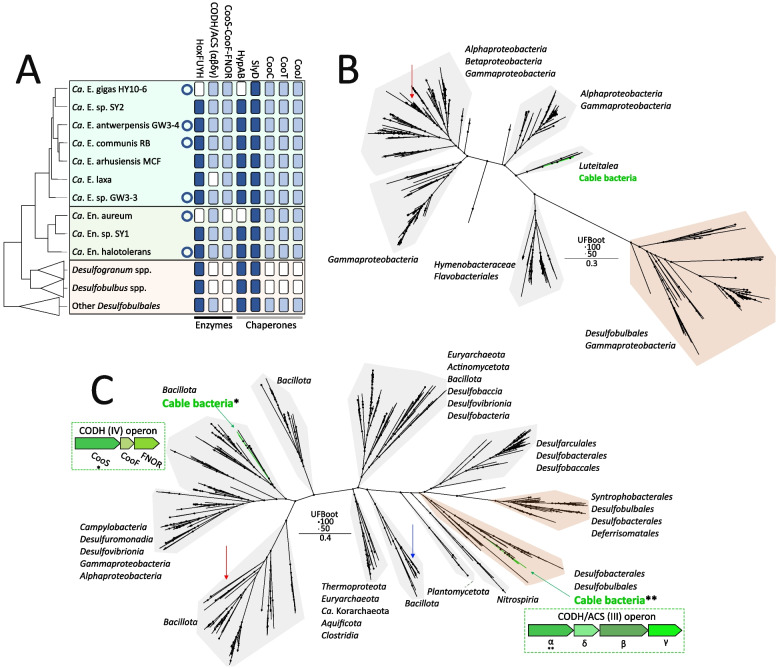
Presence and phylogeny of nickel‑binding enzymes in cable bacteria. **A** Presence/absence table of genes involved in nickel binding in cable bacteria and related organisms of the *Desulfobulbales* order. For cable bacteria, only species representatives with HQ (no circle) or complete genomes (circle) are shown. For the *Desulfobulbales* species, presence is only indicated if found in two or more genomes. **B** Maximum likelihood phylogeny of cable bacteria and other *Desulfobulbales* Ni-binding subunit sequences of the [Ni‑Fe]‑hydrogenase and RefSeq similarity search hits. Phylogeny was inferred using IQtree according to the best‑fit model (model LG + F + R8). Cable bacteria Ni-binding subunit (HoxH) sequences are indicated with green branches. Cable bacteria sequences cluster with sequences of the *Luteitalea* family. The biochemically characterized protein HoxH protein of *C. necator* is indicated with a red arrow. Black circles indicate ultrafast bootstrap values. The lowest common shared taxonomy is indicated per clade. **C** Maximum likelihood phylogeny of cable bacteria and other *Desulfobulbales* CODH protein sequences and RefSeq similarity search hits. Phylogeny was inferred using IQtree according to the best‑fit model (model LG + R9). Corresponding operons for cable bacteria are indicated, with the subunits used for the phylogeny indicated with an asterisk (CooS) or double asterisk (α subunit CODH/ACS). The biochemically characterized CooS protein sequences of *Rhodospirillum rubrum* (red arrow) and the α subunit CODH of *Moorella thermoacetica* (blue arrow) are indicated. Black circles indicate ultrafast bootstrap values. The lowest common shared taxonomy is indicated per clade

Intracellular nickel binding can play an important role in nickel homeostasis and presently, nine enzymes are known that include Ni into their reactive site [14, 15]. Our analysis indicates the putative presence of three of these known nickel‑binding enzymes in cable bacteria genomes, i.e. [Ni‑Fe]‑hydrogenase, carbon monoxide hydrogenase and acetyl‑CoA‑synthase, along with some of their accompanying accessory proteins and nickel‑binding chaperones (Fig. 3A, Table S6).

[NiFe]‑hydrogenases reversibly catalyse the conversion of molecular hydrogen into protons and electrons, and include a Ni‑Fe redox centre as the active site [90]. The [Ni‑Fe]‑hydrogenases in cable bacteria classify as a group 3d‑type, i.e., a bidirectional, heteromultimeric [Ni‑Fe]‑hydrogenase that consists of two subunits (HoxHY) which are associated with two NADH oxidoreductase‑like subunits (HoxFU) located in the cytoplasm [91, 92]. Notably, members of the *Desulfobulbales* order also encode HoxFUYH subunits, but also putatively encode several other types of [NiFe]‑hydrogenases, such as the membrane‑bound HybOCAB hydrogenase complex found in the *Desulfogranum* genus, which is not found in any cable bacteria genome (Table S6) [92]. While present in most cable bacteria genomes examined, HoxFUYH is not universally present, as the coding sequences are conspicuously absent in the closed genomes of *Ca.* E. gigas and *Ca.* En. aureum (Fig. 3A). Phylogenetic analysis of the large subunit (HoxH), which contains the catalytic site, shows a close phylogenetic relationship of HoxH in cable bacteria with those of members of the *Luteitalea* genus (phylum *Acidobacteriota*), with 67‑69% sequence identity (Fig. 3B).

[Ni‑Fe] hydrogenases require dedicated chaperone proteins for their metalation [93–96]. The chaperone proteins HypA, HypB and SlyD are putatively encoded in all cable bacteria genomes that code for the [Ni‑Fe]‑hydrogenase. In addition, a putative s*lyD* gene is found in all *Ca.* E. gigas genomes and in *Ca*. En. aureum (Fig. 3A, Table S3). However, multiple sequence alignment of SlyD in cable bacteria revealed that the histidine rich N‑terminal region is missing [95, 97], indicating loss of nickel binding properties (Fig. S9). Multiple sequence alignments of closed cable bacterium genome HypA and HypB sequences confirmed the presence of key residues involved in nickel insertion [98–100] (Fig. S10). Phylogenetic analysis shows similarity of the putative cable bacteria HypA and HypB sequences with those from the *Chloroflexota* phylum, whereas HypA and HypB sequences of other members of the *Desulfobulbales* order form a separate clade (Figs. 3C, S11, S12).

Ni‑containing enzymes of the carbon monoxide dehydrogenase (CODH) family are divided into four classes based on phylogeny and subunit composition [101–103]. Cable bacteria encode the class III and IV CODH enzymes (Fig. 3A, Table S6). Class III enzymes in bacteria are bifunctional complexes containing an acetyl‑CoA‑synthase/CODH (CODH/ACS) tetramer, and a corrinoid iron‑sulphur protein (CoFeSP, large and small subunit) dimer [104–106]. CODH/ACS plays a key role in the Wood‑Ljungdahl pathway, where CODH performs the final step of CO_2_ reduction to CO, while ACS catalyses C‑C bond formation creating the metabolic intermediate acetyl‑CoA [101, 107]. All four subunits (αβδγ) [108], are encoded in all closed genomes of cable bacteria, and at least partially in all genomes except in the genome of *Ca*. E. laxa (Fig. 3A; Table S6). No CODH/ACS subunits are found in any other of the reference genomes in *Desulfogranum* or *Desulfobulbus* genera, but they are encoded in the genus *Desulfolithobacter* and other members of the *Desulfobulbales* order (Fig. 3A; Table S6). Phylogenetic analyses indicate that the putative nickel binding subunits in cable bacteria (α and β) are related to sequences in the *Desulfobulbales* order (Fig. 3C, Fig. S13).

Class IV CODH enzymes (CooS) are monofunctional, form a homodimer, and are involved in oxidation of CO as the electron donor [109–111]. Most HQ and closed cable bacteria genomes encode a putative Class IV CODH operon coding for CooS, CooF and FNOR [112], except *Ca.* En. aureum (Fig. 3A). This operon structure is completely absent in other members of the *Desulfobulbales* order (Fig. 3A). Phylogenetic analysis of the putative CooS in cable bacteria and database hits indicate that the cable bacteria sequences cluster with those of the *Bacillota* phylum (Fig. 3C).

CODH maturation involves Ni insertion into the heteronuclear NiFe_4_S_4_ active site, which involves the accessory proteins CooC, CooT and CooJ. Genes coding for the accessory proteins CooC, CooT and CooJ are present in all HQ and closed cable bacteria genomes, as well as in genomes of the *Desulfobulbales* order, but not in the *Desulfogranum* and *Desulfobulbus* genera (Fig. 3A). CooC, CooT and CooJ sequences found in the *Desulfobulbales* order are the closest relatives to their respective homologs in cable bacteria (Fig. S14).

### The nickel export gene rcnA in cable bacteria has an expanded histidine‑rich loop

Ni export from the cytoplasm to the external environment can be mediated by direct extracellular export mechanisms such as the Resistance Nodulation Division (RND) protein family found in *Cupriavidus metallidurans* [113, 114]. Here, a three component system (CnrABC, CznABC, NccABC) embedded in both the inner and outer membrane mediates the efflux of Ni directly to the extracellular space. In our analysis, no extracellular Ni export genes were found in cable bacteria genomes, while in the closely related *Desulfogranum* genus, a putative CnrABC operon is present in two out of the three genomes (Table S6).

**Fig. 4:**
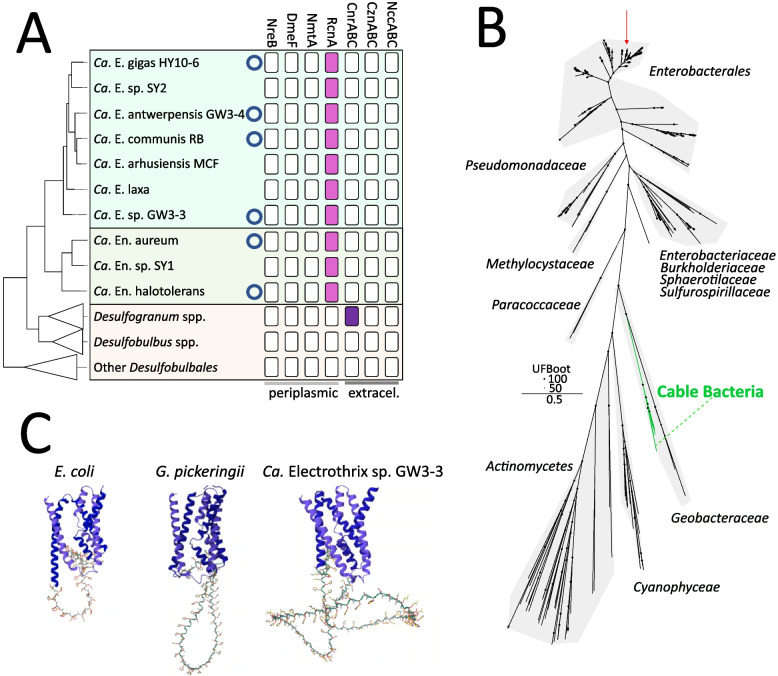
RcnA in cable bacteria. **A** Presence/absence table of nickel export genes in cable bacteria and related organisms of the *Desulfobulbales* order. Only HQ or closed (indicated with circle) genomes are shown. **B** Maximum‑likelihood phylogeny of putative cable bacteria RcnA and database (RefSeq) hits. Cable bacteria branches (green) and the closely related branches of the *Geobacteraceae* family are indicated. Black circles indicate ultrafast bootstrap values. The biochemically characterized RcnA of *E. coli* is indicated with a red arrow. The lowest common shared taxonomy is indicated per clade. **C** Alphafold2 structure predictions highlighting the expansion of the histidine‑rich loop in cable bacteria. Transmembrane helices are indicated in blue, the histidine‑rich loop is indicated in green

Alternatively, Ni export can be mediated by periplasmic nickel exporters: Ni transporters that are embedded in the inner membrane and regulate efflux of Ni to the periplasmic space. Examples of such periplasmic Ni exporters include (1) NmtA, a putative P1 type ATPase, (2) NreB, part of the major facilitator superfamily (MFS) of proteins that likely utilize the chemiosmotic gradient, (3) DmeF, part of the cation diffusion facilitator (CDF) protein family and (4) RcnA, an efflux system conveying resistance to both cobalt and nickel [115–119]. Of these genes, only a gene coding for a putative RcnA homolog is found in cable bacteria genomes, while none are found in any member of the *Desulfobulbales* order. This putative RcnA homolog is found in all closed cable bacteria genomes as well as most HQ cable bacteria genomes in our dataset (Fig. 4A). The putative RcnA protein is predicted to have canonical RcnA domain architecture, with 5 transmembrane helices (TMHs) and a central histidine‑rich loop, which is predicted to be located in the cytoplasm (TMH presence and orientation predicted with DeepTMHMM; [120]). Phylogenetic analysis shows that the cable bacteria RcnA homologs form a monophyletic clade, closely related to sequences from the *Geobacteraceae* family (Fig. 4B; 47% amino acid sequence identity with *Geobacter pickeringii* RcnA). A comparison of the protein sequences indicates that the cable bacteria RcnA is highly divergent in the histidine‑rich loop, as this loop in cable bacteria is considerably larger (100‑123 AA) and has more histidines (33‑53 histidines) than canonically described (RcnA of *Escherichia coli* 67 AA, 17 histidines) [117, 118]. Expansion of the histidine‑rich loop of RcnA in cable bacteria is visualized by prediction of the protein structure with Alphafold2 [121] in Fig. 4C.

## Discussion

**Fig. 5:**
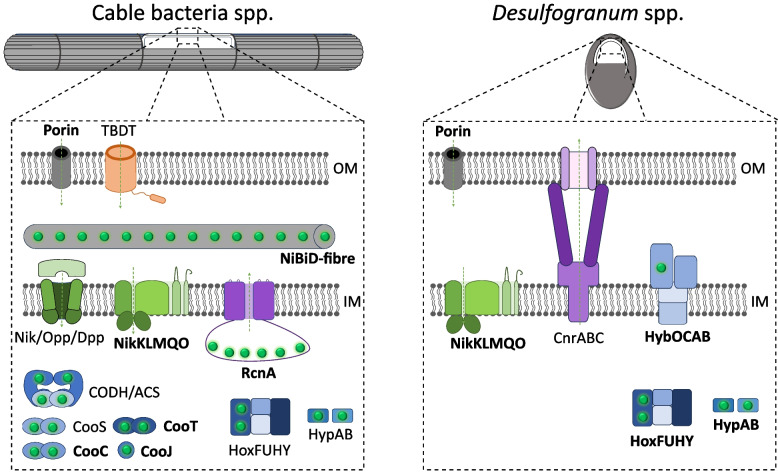
Schematic overview of Ni‑homeostasis related genes in cable bacteria and the genus. *Desulfogranum*. Putative proteins that are present in all HQ genomes are indicated in bold

Figure 5 provides an overview of putative Ni‑homeostasis genes in cable bacteria. The *Desulfogranum* genus is the closest relative of cable bacteria within the *Desulfobulbaceae* family, and encodes a very different repertoire of Ni‑homeostasis proteins (Fig. 5). Therefore, there seems to be a clear genetic adaptation to intensified Ni cycling in cable bacteria.

### Nickel import

Cable bacteria contain a conductive periplasmic fibre network that mediates electron transfer, which allows cable bacteria to oxidize sulphide in anoxic environments [1, 4, 6]. The conductive protein fibres contain a nickel cofactor not previously described in any other biological organism [11].The nickel cofactor consists of a Nickel Bis‑Dithiolene (NiBiD) complex that forms a highly conjugated planar structure [10]. Due to NiBiD incorporation in the fibre network, the periplasm has been shown to be specifically enriched in nickel [10, 11, 122]. Therefore, compared to other organisms, the acquisition of free Ni from the environment is especially important to cable bacteria, as it is needed to maintain their conductive structure (which in its turn supports their energy metabolism and growth). Dividing cells within the cable bacteria filament require Ni to extend their periplasmic fibre cage. Without sufficient Ni uptake, the fibre cage cannot be extended, and the electron transfer of the entire filament would falter.

Cable bacteria inhabit sulfidic, fine‑grained sediments, which can exhibit relatively high porewater concentrations of nickel (~ 1 µM) [123]. Therefore, it is possible that in sufficiently nickel‑rich sediments, part of the nickel import may occur by passive diffusion through porins. However, porins have a rather low permeability for cations [22, 124], while the metabolic demand for nickel is large, and so one expects that active uptake systems are in place. This idea is supported here, as cable bacteria putatively possess multiple active nickel uptake mechanisms, some of which are not found in related genomes of the *Desulfobulbales* order (Fig. 5). While the NikM(K)LQO/NikMNQO system is putatively encoded in genomes across the *Desulfobulbales* order including the cable bacteria, a putative Nik/Opp/Dpp family importer and an undefined TBDT are found exclusively in multiple cable bacteria genomes (Fig. 2A).

Phylogenetic analysis suggests that the undefined TBDT and Nik/Opp/Dpp family importer sequences of cable bacteria form separate, monophyletic clades unrelated to *Desulfobulbales* sequences (Figs. 2B, S8), which could e.g. imply lateral gene transfer to cable bacteria or gene loss in a common ancestor. Even though the NikMKLQO systems are found in genomes across the *Desulfobulbales* order, our phylogenetic and gene synteny analyses indicate that individual subunits are more similar to distantly related members of the *Desulfobulbales* order, and less similar to the more closely related *Desulfogranum* and *Desulfobulbus* species. Interestingly, it seems likely that in *Ca*. E. gigas a potential gene rearrangement of the *nikMQO* genes occurred, according to phylogenetic analysis and gene neighbourhood comparison with the *nikMKLQO* locus of other cable bacteria species (Fig. 2C). Cable bacteria of the species *Ca*. E. gigas have a large diameter (approximately 2‑10 × larger than other species of cable bacteria) and a correspondingly greater number of periplasmic fibres per unit of length [5, 42], implying a greater demand for nickel. However, the significance of *nikMQO* gene rearrangement for nickel uptake is still unclear.

### Enzymes with nickel in the reactive site

Cable bacteria putatively possess three nickel‑binding enzymes and some of their nickel‑binding associated chaperones: a cytoplasmic [NiFe]‑hydrogenase, CODH/ACS and CooS. In addition, they encode several putative intracellular nickel‑binding chaperone proteins: HypAB and CooJ. Related members in the *Desulfobulbales* order also encode several intracellular nickel‑binding proteins (Fig. 3), and thus the presence of intracellular nickel‑binding proteins alone in cable bacteria is unlikely to strongly influence the availability of free nickel compared to their close relatives. Nonetheless, there is an important difference in the type and number of intracellular nickel binding proteins within cable bacteria and their close relatives (Fig. 5).

Cable bacteria encode a putative cytoplasmic group 3d‑type [NiFe]‑hydrogenase, while other members in its order encode several other types of periplasmic or membrane‑bound [NiFe]‑hydrogenases, which cable bacteria seem to have lost (Table S6). Group 3d‑type [NiFe]‑hydrogenases are bidirectional heteromultimeric complexes of four subunits (HoxFUYH) which are located in the cytoplasm, and some have been reported to be tolerant to oxygen, such as the [Ni‑Fe] hydrogenase of *Cupriavidus necator* [125–127]. As it happens, the large HoxH subunit of [Ni‑Fe] hydrogenase in cable bacteria shares a relatively high amino acid sequence identity (55‑62%) with the HoxH subunit of *Cupriavidus necator*. Moreover, the closest phylogenetic relationship in our dataset of the HoxH subunit is not with any member of the *Desulfobulbales* order, but rather with members of the genus *Luteitalea*, which are aerobic microorganisms [128]. Together, this brings forward the hypothesis that the [Ni‑Fe]‑hydrogenase found in cable bacteria could also be oxygen tolerant. This would be congruous with the fact that part of the cable bacteria filament is located within high‑oxygen conditions in the oxic zone of the sediment [4, 9, 129].

CODH/ACS plays a major role in the Wood‑Ljungdahl pathway of carbon fixation [105–107]. Previous genome analysis suggested that autotrophic CO_2_ fixation in cable bacteria occurs via the Wood‑Ljungdahl pathway [18]. Furthermore, isotope labelling experiments have shown that cable bacteria are capable of autotrophic CO_2_ fixation [9]. Although present in almost all cable bacteria, CODH/ACS coding genes are not found in members of the genera *Desulfogranum* and *Desulfobulbus*, and the protein sequences are phylogenetically related to other members within the *Desulfobulbales* order (Fig. 3AC), e.g. chemolithoautotrophic *Desulfomarina profundi* [125]. In addition, the CODH/ACS coding genes are found in the related *Desulfolithobacter dissulfuricans* genome (Fig. 1, 3C), which could suggest a loss of the Wood-Ljungdahl pathway in the *Desulfogranum* and *Desulfobulbus* genera. [130].

In addition to CODH/ACS, most cable bacteria genomes possess a *cooS‑cooF‑*FNOR gene cluster, except for *Ca*. En. aureum. This cluster is not found in other members of the *Desulfobulbales* order and seems to be a unique genetic adaptation in cable bacteria, with their CooS sequences related to sequences found in the *Bacillota* phylum (Fig. 4C). In *Geobacter sulfurreducens*, CooS, CooF (an iron‑sulphur protein) and FNOR (FAD‑dependent NAD(P)H oxidoreductase) were inferred to be involved in growth with carbon monoxide as electron donor [112]. The *cooS‑cooF‑*FNOR gene cluster in *G. sulfurreducens* also contains *rcoM*, encoding a CO‑sensing transcriptional regulator [112, 130], which however appears to be absent in cable bacteria genomes. Whether cable bacteria can utilize carbon monoxide for energy conservation is yet to be studied experimentally.

### Nickel export

Excess nickel is toxic [28], so most organisms incorporate an export mechanism that can deal with superfluous nickel [131]. Extracellular Ni export is mediated through members of the Resistance Nodulation Division (RND), like CnrABC, NccABC and CznABC [113]. Indeed, several members of the *Desulfogranum* genus, whom cable bacteria share the closest common ancestor with, contain a cluster homologous to the *cnrABC* operon found in the nickel‑resistant *Cupriavidus* metallidurans [131, 132]. None of the HQ cable bacteria genomes feature this *cnrABC* operon (Fig. 2A), thus it is a possibility that their common ancestor lost it. Experimental evidence suggests that periplasmic Ni concentrations lead to a signal transduction chain resulting in transcription initiation of *cnr* promoters, controlling the expression of the cnrABC efflux pump, and therefore it has been suggested that RND family proteins mainly export periplasmic Ni [114]. In this context, and as cable bacteria may be presumed to have a strong requirement for Ni in the periplasm, it is perhaps unsurprising that the genes coding for the CnrABC efflux pump are not present in cable bacteria. Thus, as has been suggested for other organisms without an identifiable outer membrane nickel efflux system [131], cable bacteria could prevent excess Ni(II) by carefully balanced uptake and utilization of nickel.

A putative RcnA homolog for the export of nickel from the cytoplasm to the periplasm is encoded in all closed genomes and most HQ genomes. This gene is likely universally present in all cable bacteria, and we speculate that it is absent in some genomes due to genome incompleteness. As the RcnA homolog is not found in any other member of the *Desulfobulbales* order, it seems to be a specific genetic adaptation in cable bacteria, with the gene perhaps acquired through lateral gene transfer (Fig. 4B). This putative lateral gene transfer event likely occurred a single time to the common ancestor of cable bacteria, as the cable bacteria RcnA sequences form a monophyletic clade with related database hits (Fig. 4B). This exclusive possession of *rcnA* in the cable bacteria genomes suggests a physiological need for cable bacteria to export nickel to the periplasm, unlike any other member of the *Desulfobulbales* order. This would fit with the incorporation of a unique nickel cofactor into the periplasmic conductive fibres that are only present in cable bacteria [10, 11], although the cellular localization of the biosynthetic machinery for nickel cofactor production has not yet been elucidated. Moreover, the exact mechanism by which RcnA conveys nickel resistance in *E. coli* is not fully understood, as it is thought that this protein could function as a nickel exporter or a chelator using its histidine‑rich loop [118]. The genetic expansion of the histidine rich loop in cable bacteria RcnA sequences is found in all cable bacteria genomes and might be related to the synthesis of the unique nickel cofactor found in cable bacteria [10, 11]. Large cytoplasmic vesicles have been observed in cable bacteria with cryogenic electron tomography, and it has been suggested that these vesicles contain conductive fibre components [122]. We speculate that cable bacteria RcnA could accommodate production and incorporation of the nickel‑cofactor in conductive fibre components within these cytoplasmic vesicles, by nickel chelating/chaperone activity and thus locally concentrating Ni(II) within the vesicles upon release.

## Conclusions

In this study, we demonstrated that cable bacteria have uniquely adapted their genetic repertoire for nickel homeostasis compared to closely related family members. In addition, we improved the current cable bacteria genome information by generating two closed genomes, one of which represents a new species. We showed the presence of a unique RcnA homolog in cable bacteria, which contains a histidine‑rich loop that has been notably expanded compared to RcnA homologs found in other organisms. For nickel import proteins and nickel‑binding proteins, we observed inter‑species diversity in cable bacteria, and showed that the genes coding for all these protein (complexes) were either not found in the *Desulfobulbales* order and phylogenetically related to distant taxa, or when found within the *Desulfobulbales* order, unrelated to the closely related genera of *Desulfogranum* and *Desulfobulbus*. All‑in‑all, these results are congruent with the recent finding of a novel nickel‑containing cofactor that is used by cable bacteria for long distance electron conduction.

### Description of *Candidatus* Electrothrix antwerpensis sp. nov.

*Candidatus* Electrothrix antwerpensis (ant.wer.pen’sis, from L. adj. antwerpensis, pertaining to the location where the strain was obtained in a clonal enrichment culture, Antwerpen (Belgium); N.L. fem. adj. antwerpensis). This taxon is represented by strain GW3‑4.Growth is filamentous, with individual cell size ranging from 0.4–0.5 µm in diameter and 3.4–6.3 µm in length. Filaments exhibit the distinct features of cable bacteria. The outer surface shows a pattern of parallel ridges, which contain the periplasmic conductive fibres (*N* = 23 ± 1). Raman microscopy confirmed the presence of the two prominent frequency bands at 373 and 492 cm^−1^ indicative of the NiBiD cofactor exclusively found in cable bacteria. Growth by electrogenic sulphur oxidation with oxygen as electron acceptor in salt water conditions. In addition, the genome content suggests sulphide oxidation may also be coupled to DNRA. The complete protologue can be found in Table S7.

### Supplementary Information

Supplementary Material 1.

Supplementary Material 2: Table S1.

Supplementary Material 3: Table S6.

Supplementary Material 4; Table S8.

## Data Availability

Sequencing data for this study has been deposited at the National Center for Biotechnology Information (NCBI) under BioProject ID PRJNA1081657. Genbank files for the Candidatus Electrothrix antwerpensis GW3‑4 genome (GCA_037902255.1) and Candidatus Electrothrix gigas HY10‑6 genome (GCA_037901825.1) are available.
