# Supplementary material for: Comparative genomic analysis of nickel homeostasis in cable bacteria
Source: BMC Genomics. 2024 Jul 15;25:692. doi: 10.1186/s12864-024-10594-7 (PMC11247825; doi:10.1186/s12864-024-10594-7)
Supplement: Supplementary file 1 — Supplementary Material 1. [file 12864_2024_10594_MOESM1_ESM.docx]

*Supplementary Material*

# Comparative genomic analysis of nickel homeostasis in cable bacteria

**Anwar Hiralal^1^, Jeanine S. Geelhoed^1^, Sinje Neukirchen^1^ and Filip J.R. Meysman^1,2^**

1. Geobiology research group, University of Antwerp, Antwerp, Belgium.
2. Department of Biotechnology, Delft University of Technology, Delft, The Netherlands.

corresponding author: filip.meysman@uantwerpen.be; F.J.R.Meysman@tudelft.nl

**Table S1:** Overview of dataset quality and genome accession numbers

*Table S1 is available as a separate Excel file.*

**Table S2:** Nanopore sequencing overview

|  | GW3-4 | HY10-6 |
| --- | --- | --- |
| Nanopore flow cell | FLO-MIN106 | FLO-MIN106 |
| Sequencing kit | SQK-LSK109 | SQK-LSK109 |
| Barcoding kit | EXPNBD114 | EXPNBD114 |
| Raw bases (bp) | 6,165,653,830 | 5,232,155,572 |
| Raw reads | 3,133,217 | 3,050,591 |
| Bases after QC (bp) | 6,068,787,047 | 5,145,899,861 |
| Reads after QC | 3,131,882 | 3,042,377 |
| Average read length (bp) | 1,967 | 1,715 |
| Read length N50 (bp) | 3,041 | 2,649 |

**Table S3:** Illumina MiSeq sequencing overview

|  | GW3-4 | HY10-6 |
| --- | --- | --- |
| Sequencing platform | Illumina HiSeq | Illumina HiSeq |
| Paired raw bases | 3,341,656,878 | 4,121,670,028 |
| Paired raw reads | 11,065,089 | 13,647,914 |
| Paired bases after QC (bp) | 1,88,332,8696 | 2,275,235,282 |
| Paired reads after QC | 6,773,745 | 8,166,312 |
| Average insert size (bp) | 254 | 253 |

**Table S4:** Genome features of *Ca*. Electrothrix antwerpensis strain GW3‑4 and *Ca*. Electrothrix gigas strain HY10‑6

| Name | *Ca.* Electrothrix antwerpensis  strain GW3‑4 | *Ca.* Electrothrix gigas  strain HY10‑6 |
| --- | --- | --- |
| Genome size | 4,642,691 | 3,571,595 |
| Genome structure | circular | circular |
| Coding sequences | 4,215 | 3,341 |
| Protein coding genes | 4025 | 3107 |
| 16S‑23S‑5S rRNA loci | 2 | 2 |
| GC content (%) | 49.5 | 46.0 |
| Assembly coverage | 42X | 33X |

**Table S5:** ANI table of cable bacteria genomes

**
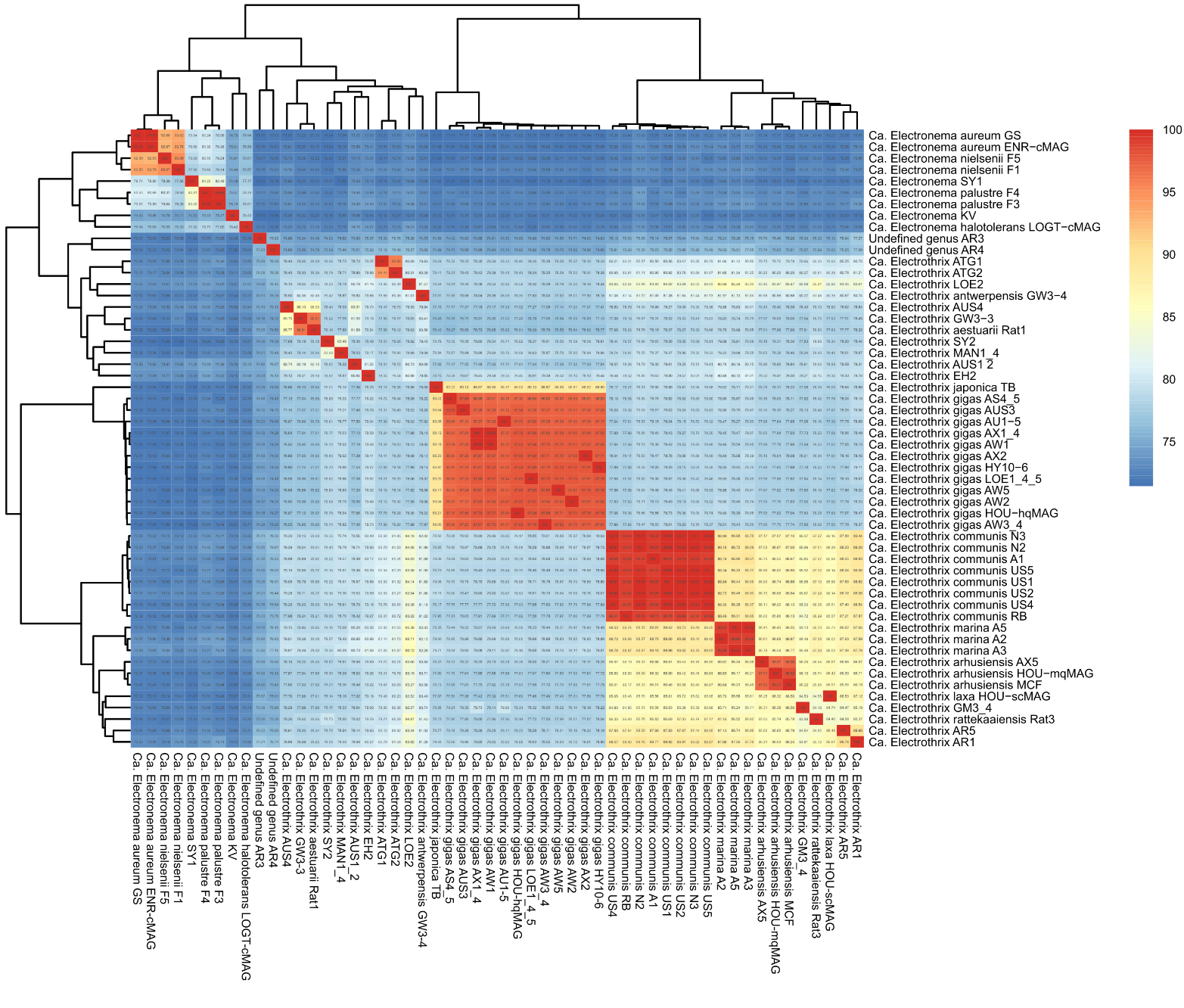
**

**Table S6:** locus tags of Ni-homeostasis genes in the cable bacteria dataset

*Table S6 is available as a separate Excel file.*

**Table S7:** Protologue table for *Candidatus* Electrothrix antwerpensis.

| **Species name** | *Candidatus* Electrothrix antwerpensis |
| --- | --- |
| **Genus name** | *Candidatus* Electrothrix |
| **Specific epithet** | antwerpensis |
| **Type strain** | GW3-4 |
| **Type species of the genus** | *Candidatus* Electrothrix arhusiensis |
| **Genus status** | Candidatus |
| **Species etymology** | Description of *Candidatus* Electrothrix antwerpensis sp. nov.: “ant.wer.pen’sis, from L. adj. antwerpensis, pertaining to the location where the strain was obtained in a clonal enrichment culture, Antwerpen (Belgium); |
| **Species status** | sp. nov. |
| **Assembly project** | PRJNA1081657 |
| **Genome accession number** | GCA_037902255.1 |
| **Genome topology** | Circular |
| **Genome Size (bp)** | 4,642,691 |
| **GC%** | 49.5 |
| **Country of origin** | The Netherlands |
| **Region of origin** | Zeeland |
| **Sample source** | Salt marsh sediment |
| **Geographical location** | Rattekaai (Scheldt-Rhine delta) |
| **Latitude** | 51.439051°N |
| **Longitude** | 4.168504°E |
| **Sample Depth** | -15-0 cm |
| **Assembly method** | Hybrid |
| **Sequencing technology** | Oxford Nanopore R9.4.1 and Illumina HiSeq |
| **Binning** | Not performed |
| **Assembly software used** | Flye 1.2 |
| **Habitat** | Intertidal zone (coastal habitat) |
| **Miscellaneous, extraordinary features relevant for the description** | Circular genome obtained from a metagenomic sample of a clonal cable bacterium enrichment |

**Table S8:** locus tags of general metabolism genes in cable bacteria closed genomes

*Table S8 is available as a separate Excel file.*


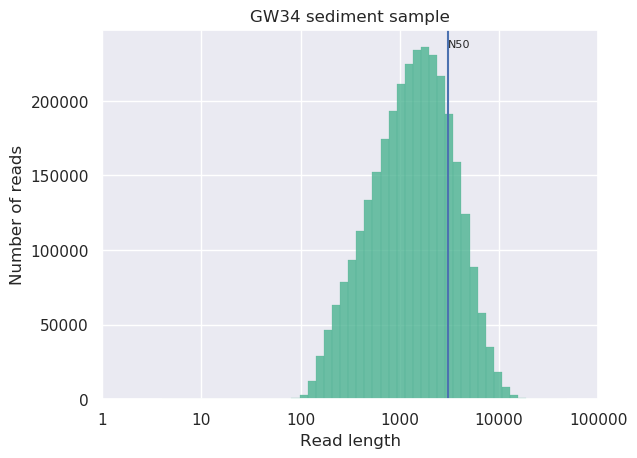

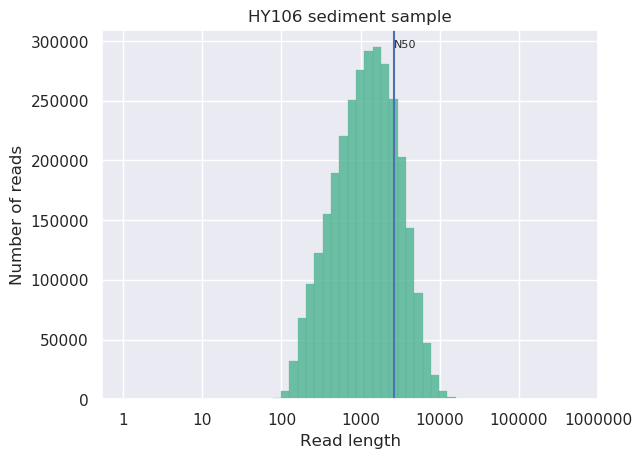


**Figure S1:** Read length distribution of nanopore reads. Left: log-scaled read length histogram of the GW3-4 sediment sample. Right: log-scaled read length histogram of the HY10-6 sediment sample. Figures obtained using NanoPlot (De Coster et al., 2018).

**
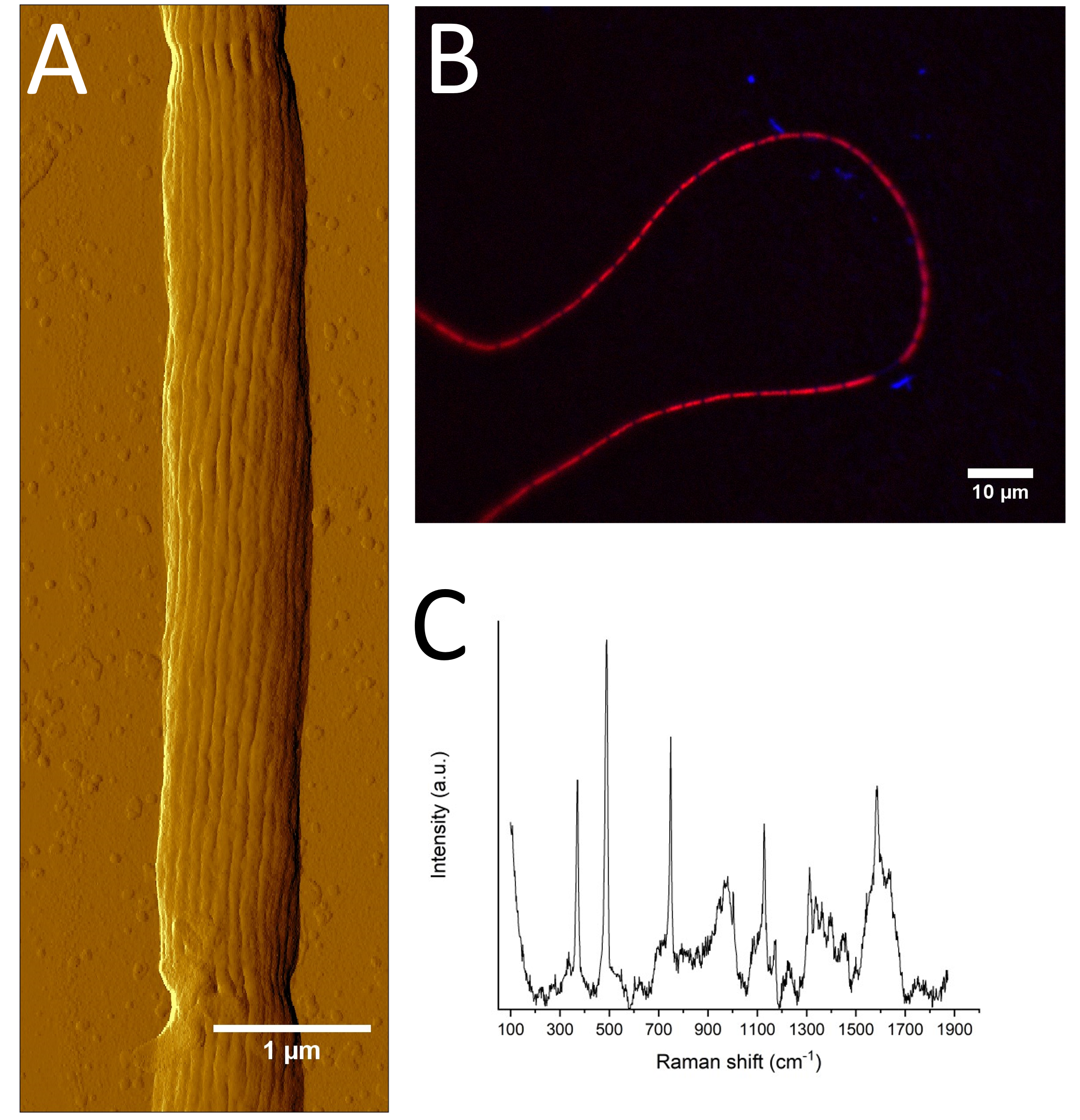
**

**Figure S2: Characterization of *Candidatus* Electrothrix antwerpensis GW3-4. A**) Atomic Force Microscopy on an intact filament. The characteristic ridge structure found in cable bacteria is visible (Cornelissen et al., 2018; Pfeffer et al., 2012). Individual cells (N=16) were measured for their dimensions and ridge numbers were counted. Ridge numbers were calculated by multiplying the visible ridges (11-12) by 2 as only half of the filament is visible. Cell width was calculated from the observed width and height of each cell (√(W/H)), as the cells are flattened to an elliptical shape **B**) Fluorescence *in situ* hybridization with probe DSB706 (Cy5, red), targeting *Desulfobulbaceae* (Loy et al., 2002; Lücker et al., 2007). The filamentous morphology is visible. **C**) Raman spectrum of an intact filament collected with a green laser (532 nm). The low frequency bands at 373 and 492 cm^‑1^ are indicative for the presence of a sulfur ligated nickel cofactor exclusively found in cable bacteria (Boschker et al., 2021; Smets et al., 2024).

**
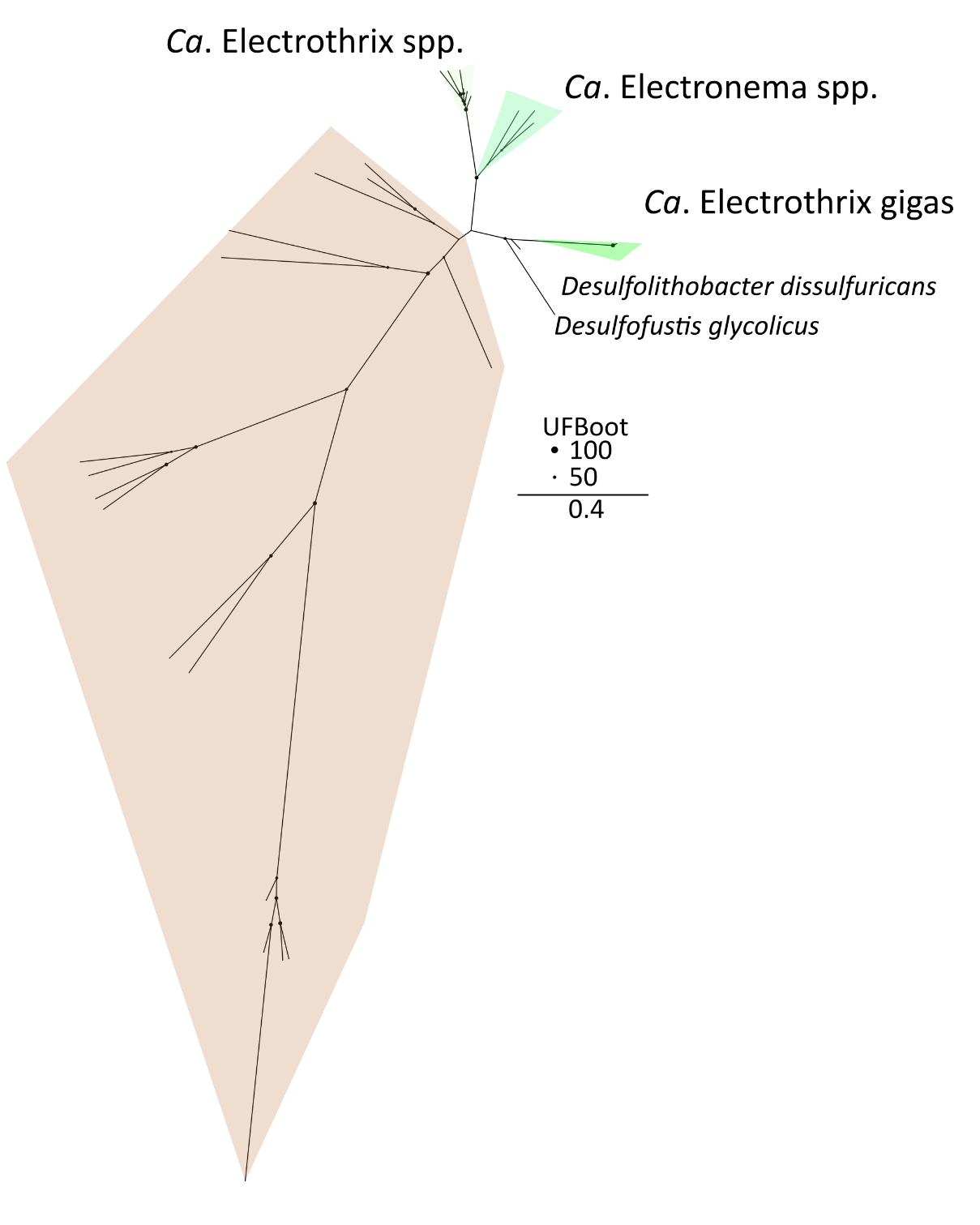
**

**Figure S3:** **Maximum-likelihood phylogeny of *nikM* homologs in cable bacteria and the *Desulfobulbales* order.** Phylogeny inferred using IQtree (Nguyen et al., 2015) according to the best fit model (model Q.pfam+I+G4). Cable bacteria NikM homolog clades are indicated (green). NikM sequences from *Ca*. E. gigas do not cluster with sequences from other *Ca*. Electrothrix species.

**
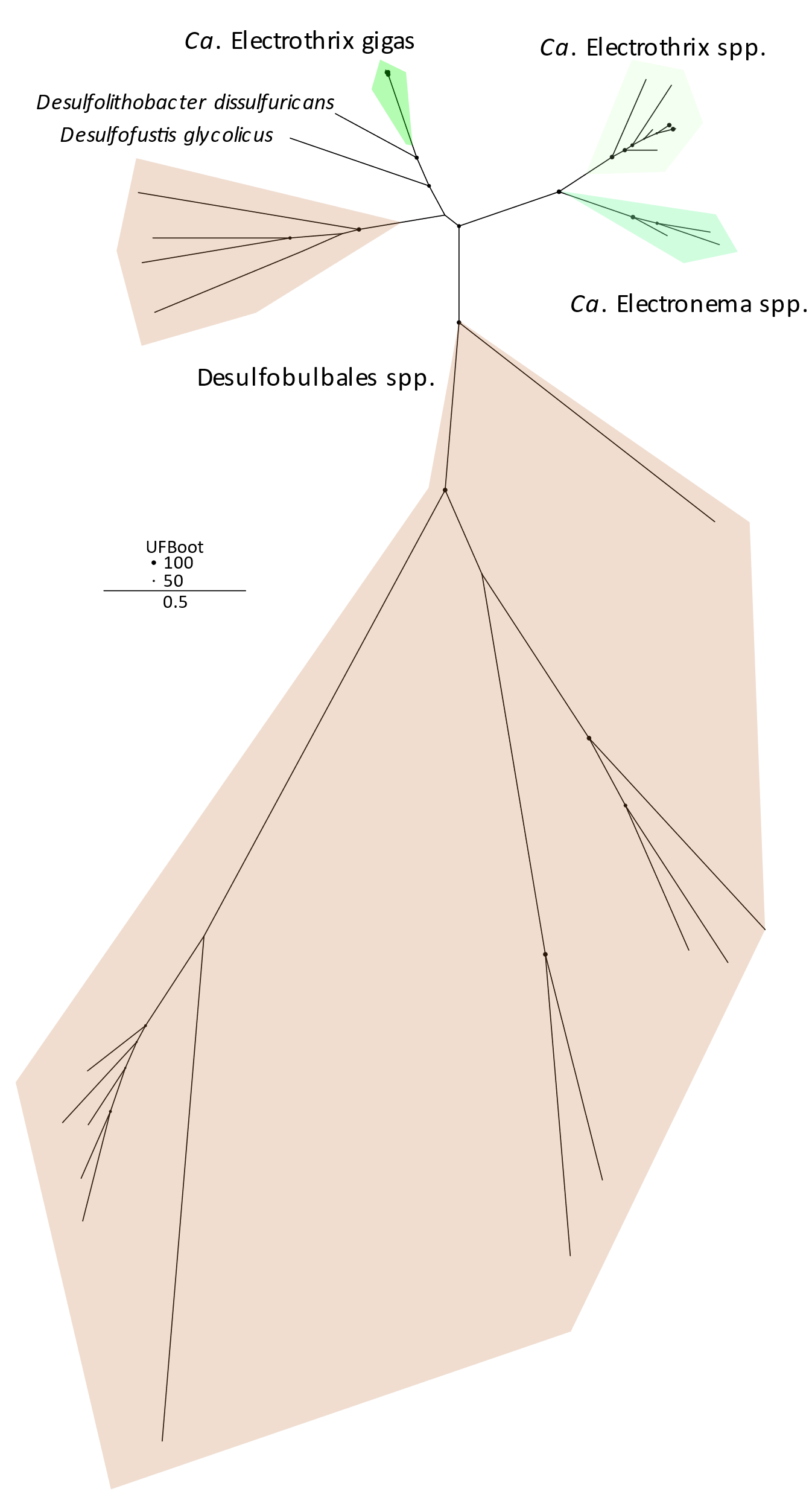
**

**Figure S4: Maximum-likelihood phylogeny of *nikQ* homologs in cable bacteria and the *Desulfobulbales* order.** Phylogeny inferred using IQtree (Nguyen et al., 2015) according to the best fit model (model Q.pfam+I+G4). Cable bacteria NikQ homolog clades are indicated (green). NikQ sequences from *Ca*. E. gigas do not cluster with sequences from other *Ca*. Electrothrix species.


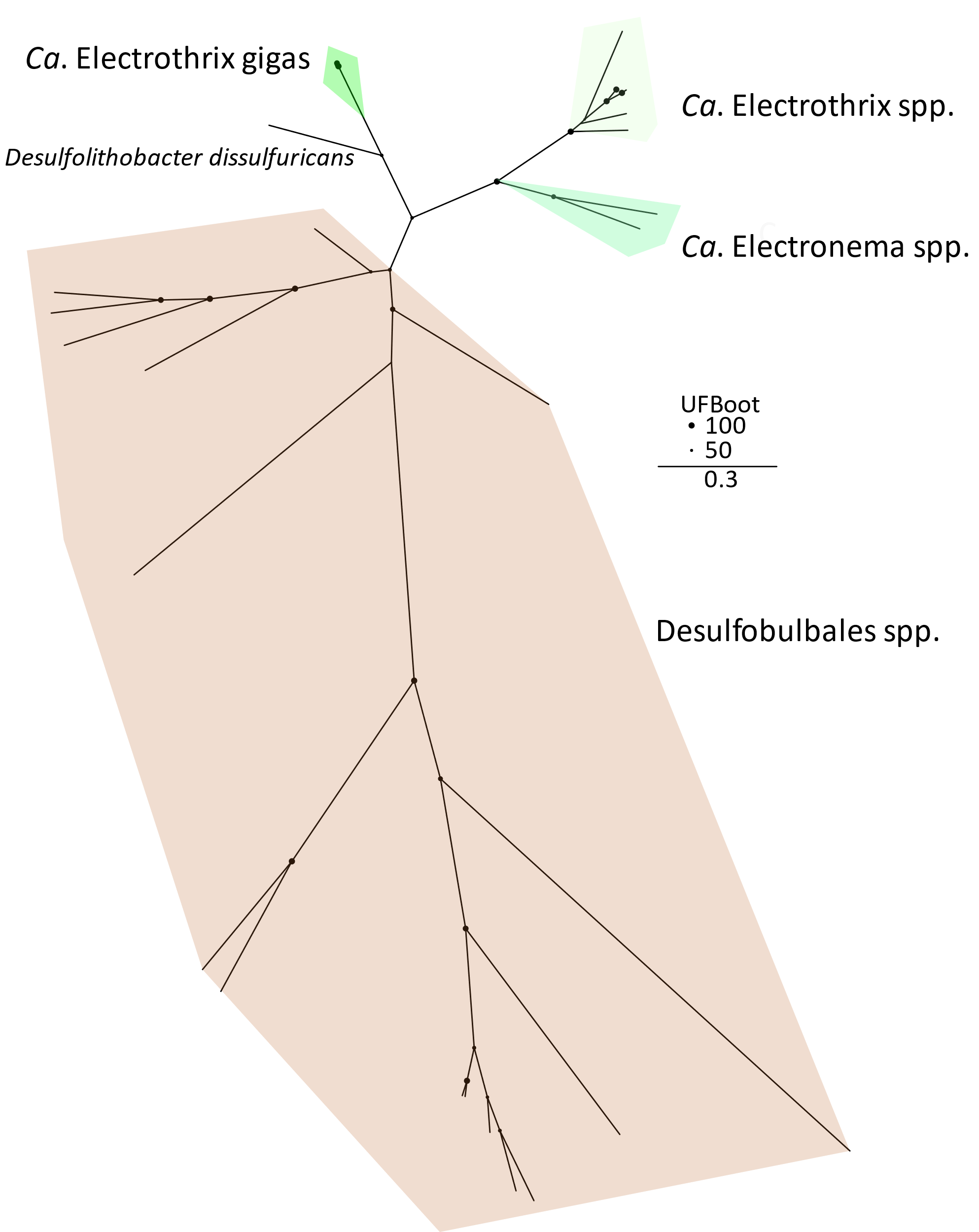


**Figure S5: Maximum-likelihood phylogeny of *nikO* homologs in cable bacteria in cable bacteria and the *Desulfobulbales* order.** Phylogeny inferred using IQtree (Nguyen et al., 2015) according to the best fit model (model Q.pfam+I+G4). Cable bacteria NikO homolog clades are indicated (green). NikO sequences from *Ca*. E. gigas do not cluster with sequences from other *Ca*. Electrothrix species.


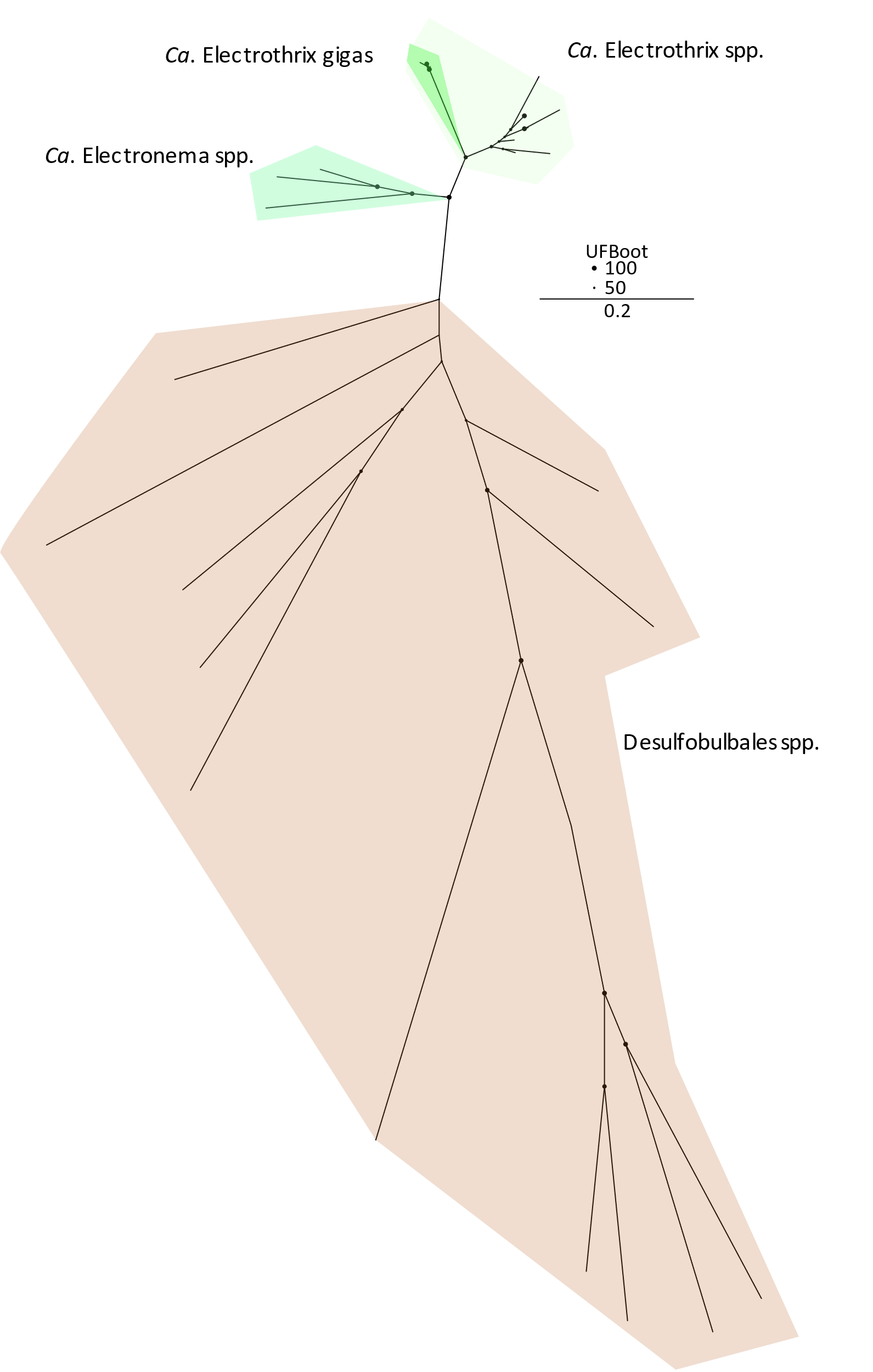


**Figure S6: Maximum-likelihood phylogeny of *nikK* homologs in cable bacteria and the *Desulfobulbales* order.** Phylogeny inferred using IQtree (Nguyen et al., 2015) according to the best fit model (model Q.pfam+I+G4). Cable bacteria NikK homolog clades are indicated (green). NikK sequences from *Ca*. E. gigas cluster with sequences from other *Ca*. Electrothrix species.


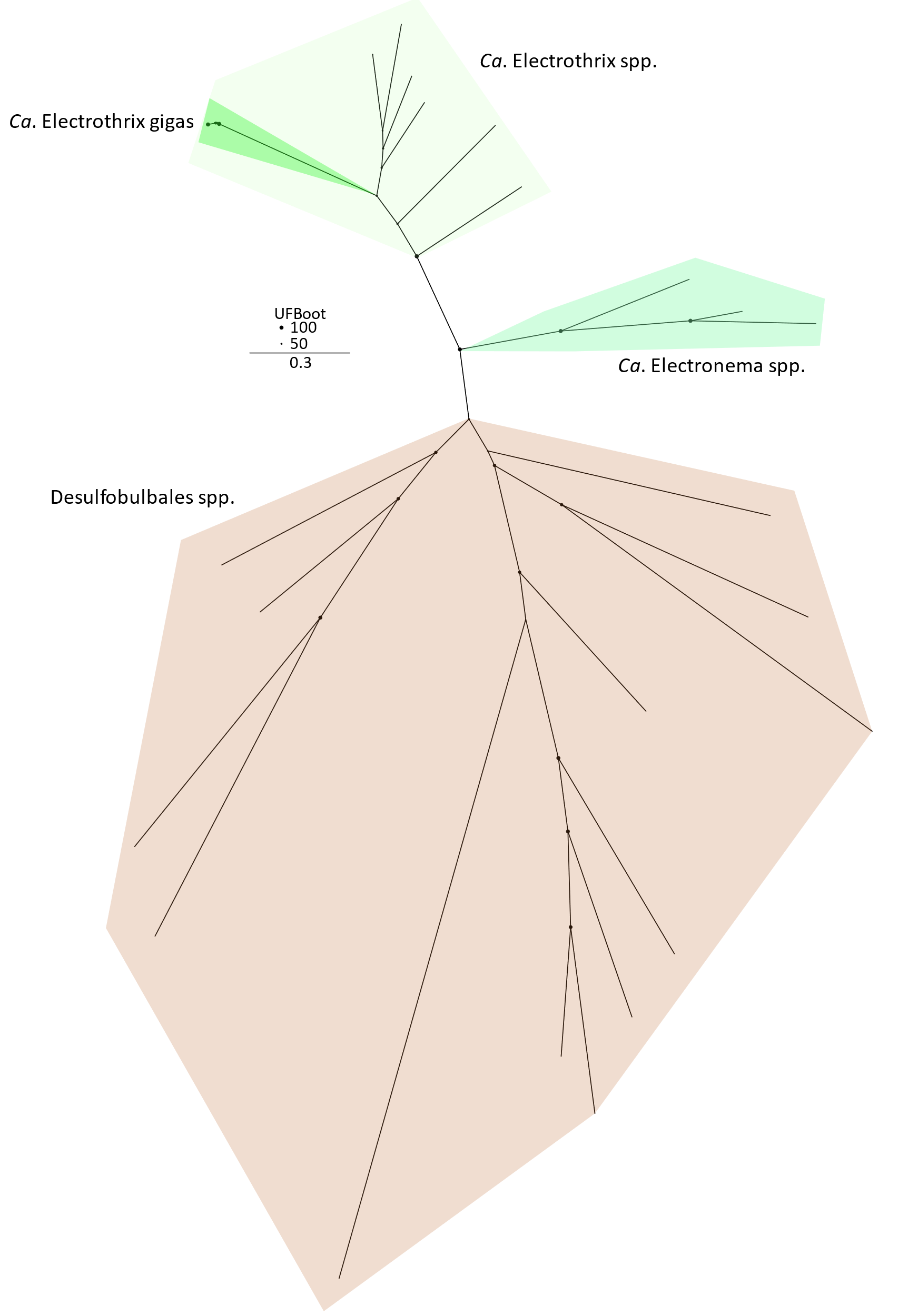


**Figure S7: Maximum-likelihood phylogeny of *nikL* homologs in cable bacteria and the *Desulfobulbales* order.** Phylogeny inferred using IQtree (Nguyen et al., 2015) according to the best fit model (model Q.pfam+I+G4). Cable bacteria NikL homolog clades are indicated (green). NikL sequences from *Ca*. E. gigas cluster with sequences from other *Ca*. Electrothrix species.


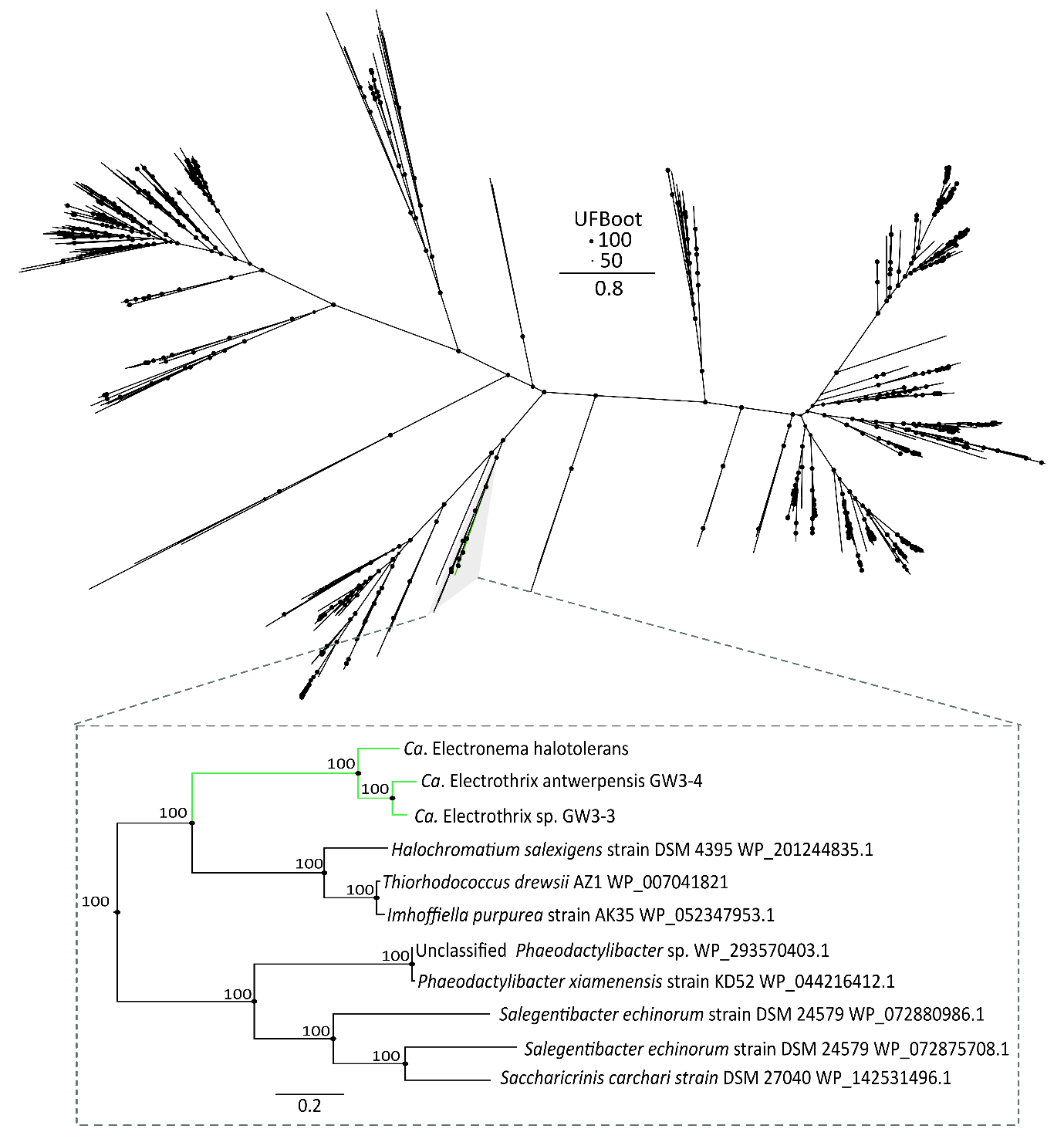


**Figure S8: Maximum-likelihood phylogeny of TBDT homologs found upstream of the *nikABCDE* operon in cable bacteria, and RefSeq database hits.** Phylogeny inferred using IQtree (Nguyen et al., 2015) according to the best fit model (model LG+F+R10). Cable bacteria TBDT homolog sequences (green branches) cluster with sequences found in species of the *Chromatiaceae* family (see insert box).

**
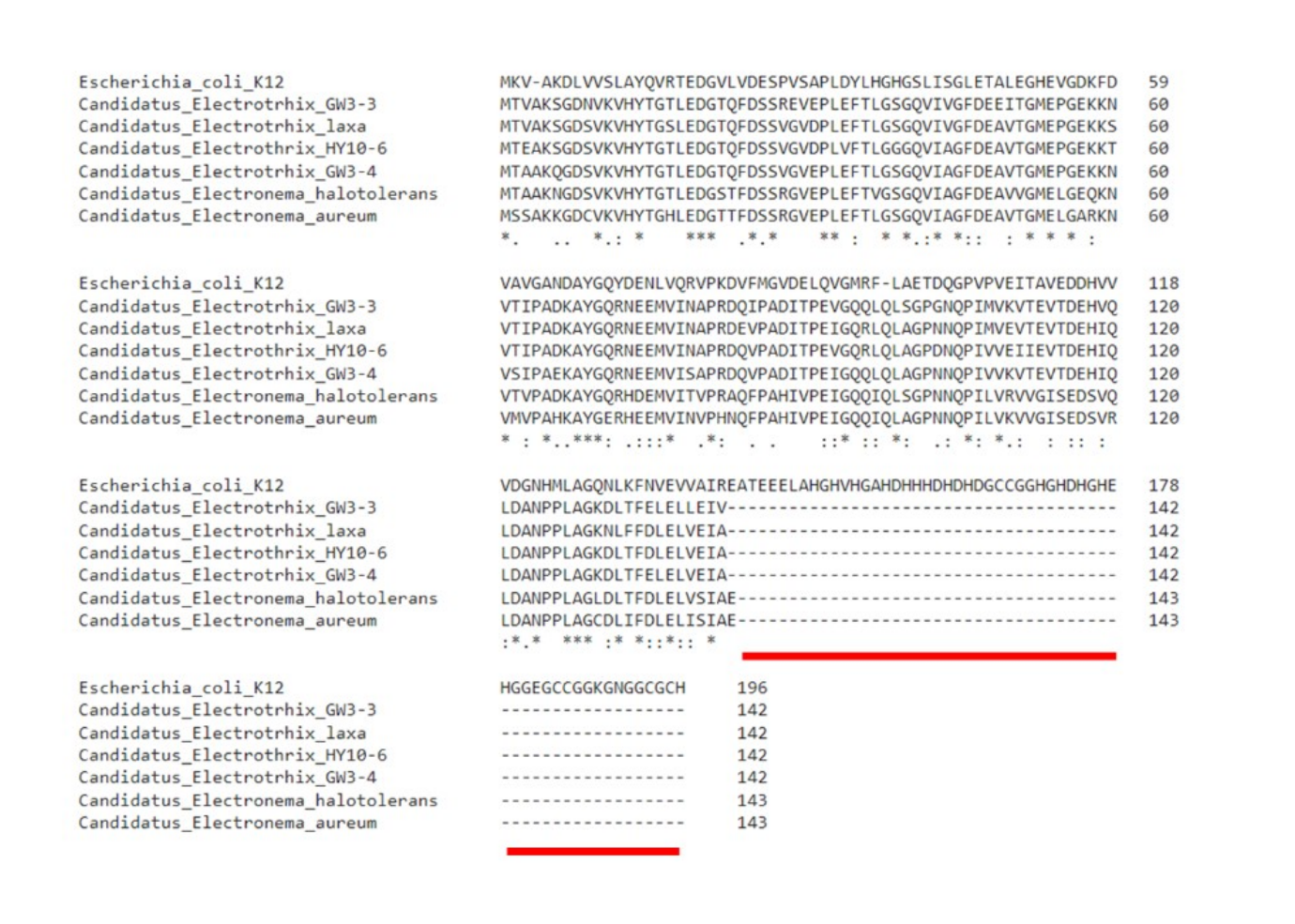
**

**Figure S9: C-terminal end is missing in Cable bacteria SlyD**. Multiple sequence alignment of selected SlyD sequences of cable bacteria and the biochemically characterized SlyD of *Escherichia coli*. The C-terminal end is missing 53-54 amino acids (red), which are crucial for nickel binding (Kaluarachchi et al., 2009; Wülfing & Plückthun, 1994).


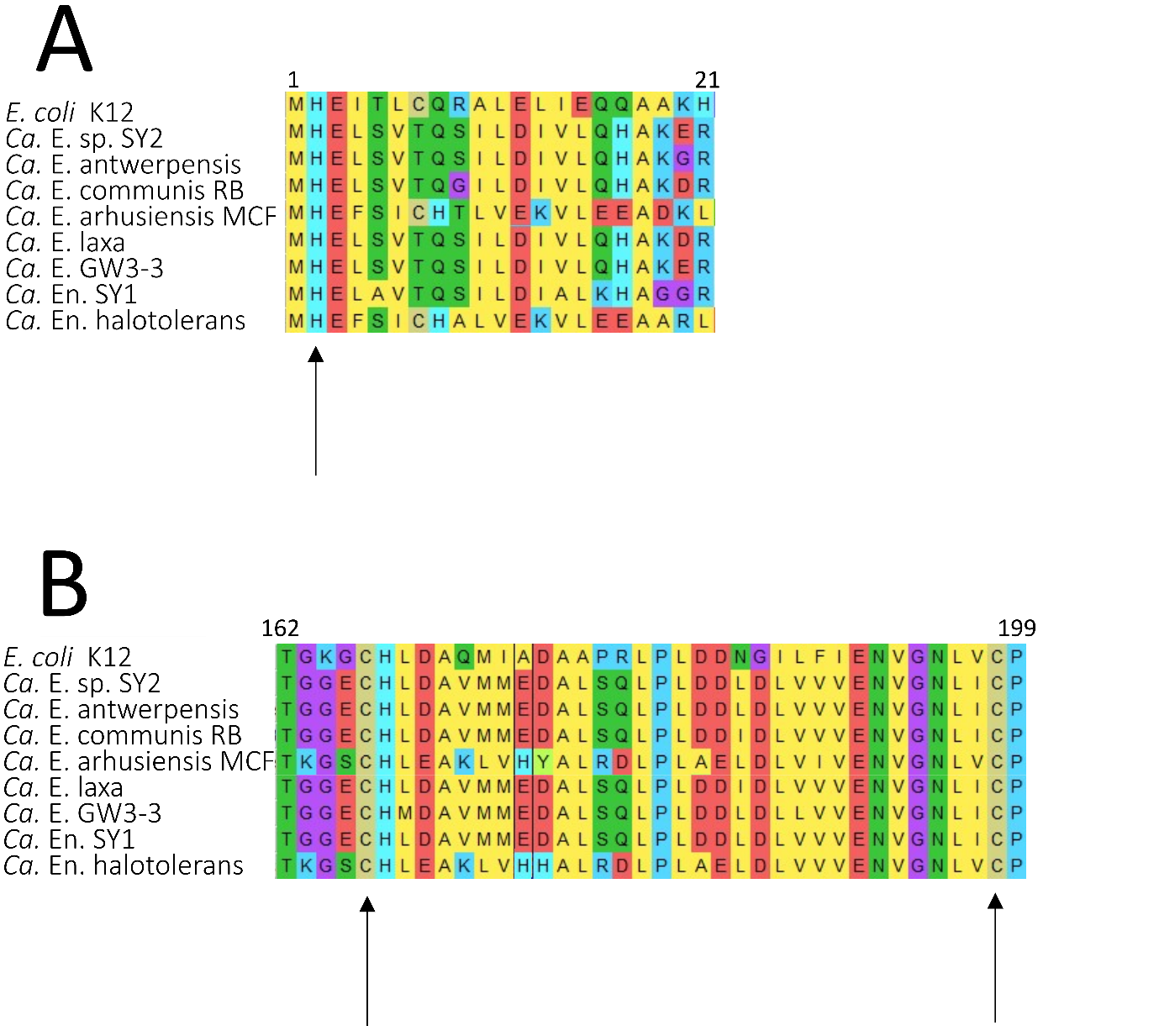


**Figure S10. Multiple sequence alignment of HypA and HypB sequences**. **A)** HypA MSA. Crucial nickel insertion residue (arrow) is indicated (Lacasse & Zamble, 2016) **B)** HypB MSA. Two nickel insertion residues are indicated (arrow) (Leach et al., 2005).


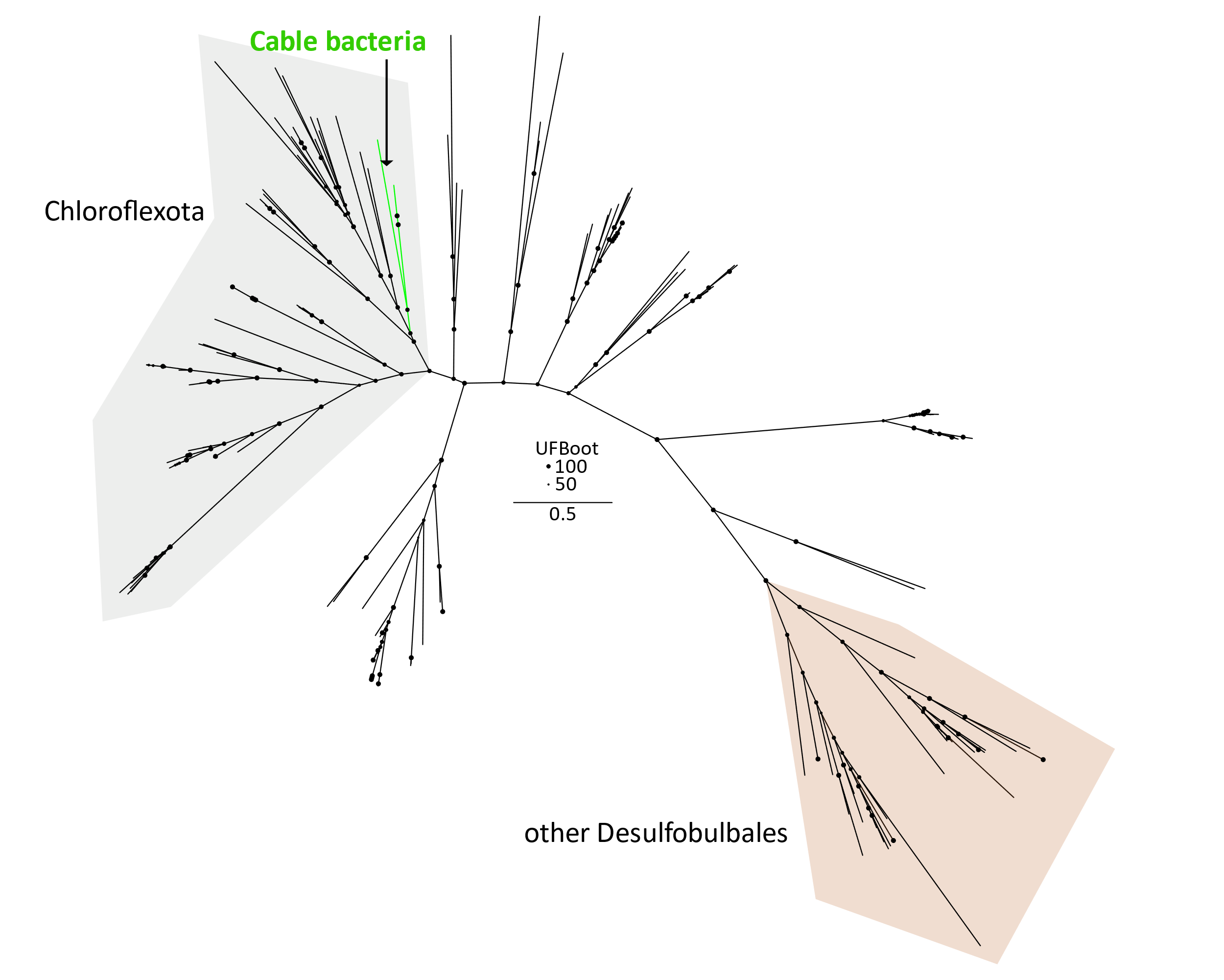


**Figure S11: Maximum likelihood phylogeny of cable bacteria and other *Desulfobulbales* HypA protein sequences and RefSeq similarity search hits.** Phylogeny inferred using IQtree (Nguyen et al., 2015) according to the best fit model (model LG+R9). Clusters are indicated. Green branches correspond to cable bacteria HypA sequences. Black circles indicate ultrafast bootstrap values.


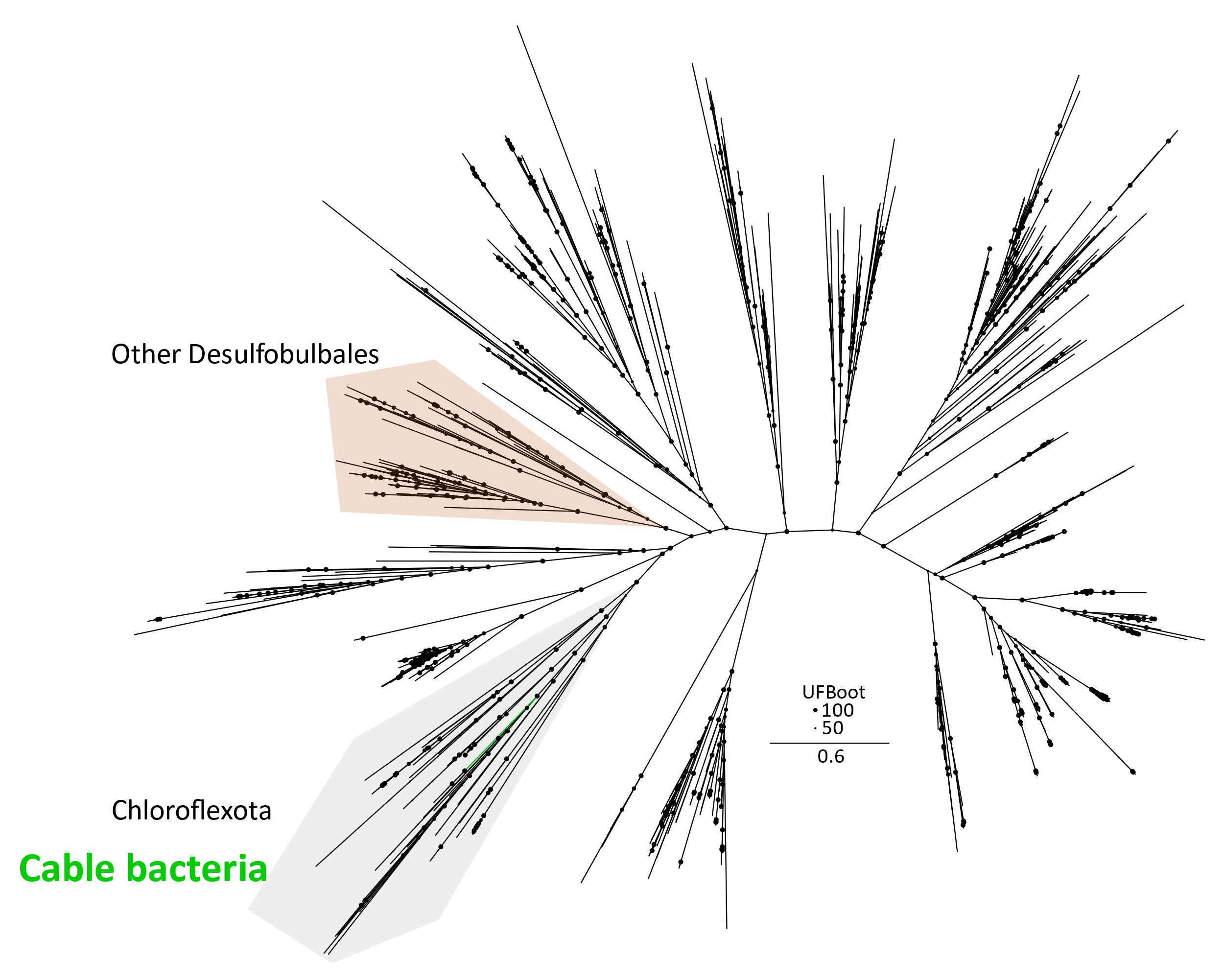


**Figure S12: Maximum likelihood phylogeny of cable bacteria and other Desulfobulbales HypB protein sequences and RefSeq similarity search hits.** Phylogeny inferred using IQtree (Nguyen et al., 2015) according to the best fit model (model LG+R9). Clusters are indicated. Green branches correspond to cable bacteria HypB sequences. Black circles indicate ultrafast bootstrap values.


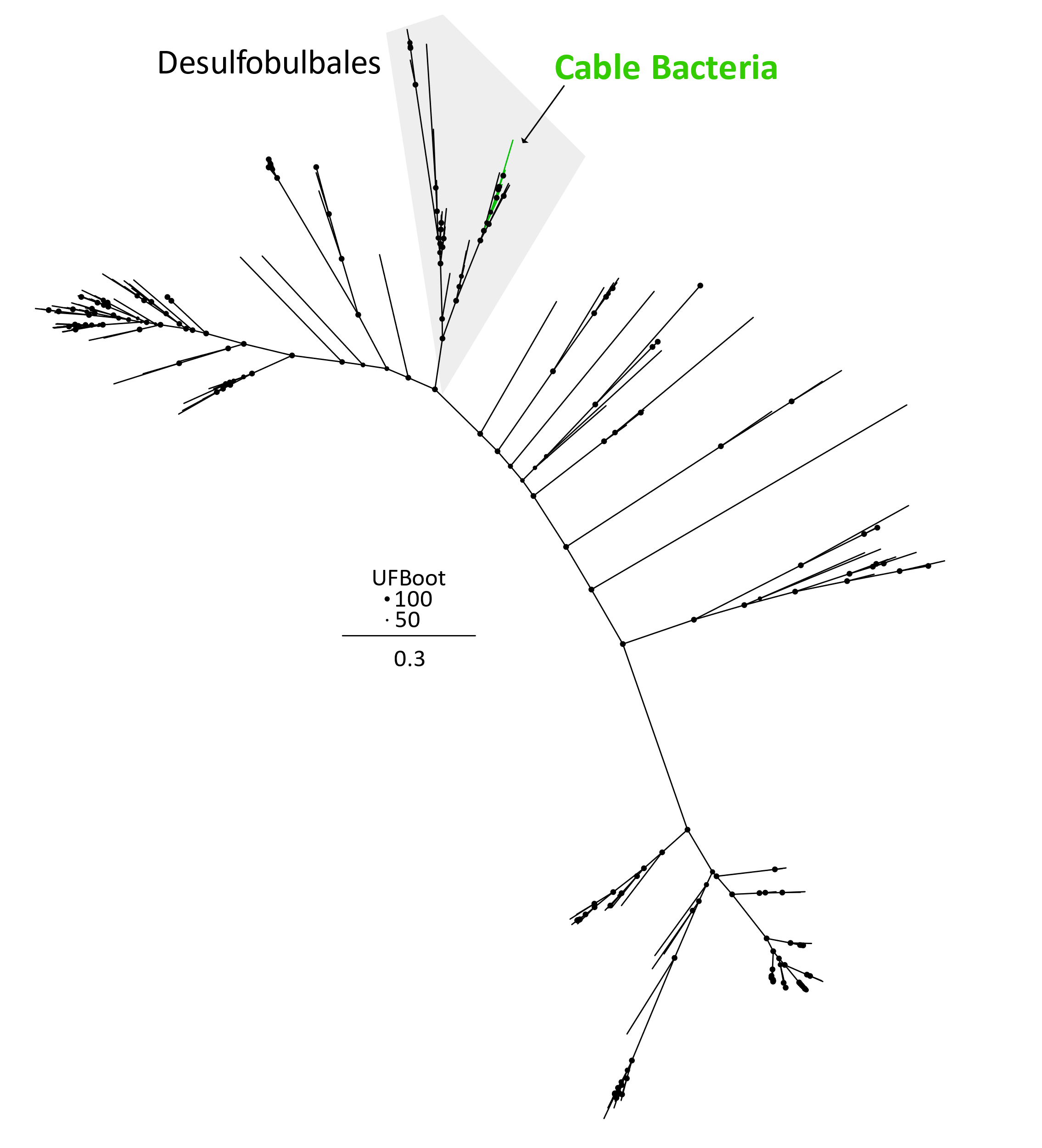


**Figure S13: Maximum likelihood phylogeny of cable bacteria and other *Desulfobulbales* ACS/CODH beta subunit sequences and RefSeq similarity search hits.** Phylogeny inferred using IQtree (Nguyen et al., 2015) according to the best fit model (model LG+F+R6). Green branches correspond to cable bacteria sequences. The grey cluster exclusively contains sequences of the Desulfobulbales order. Black circles indicate ultrafast bootstrap values.


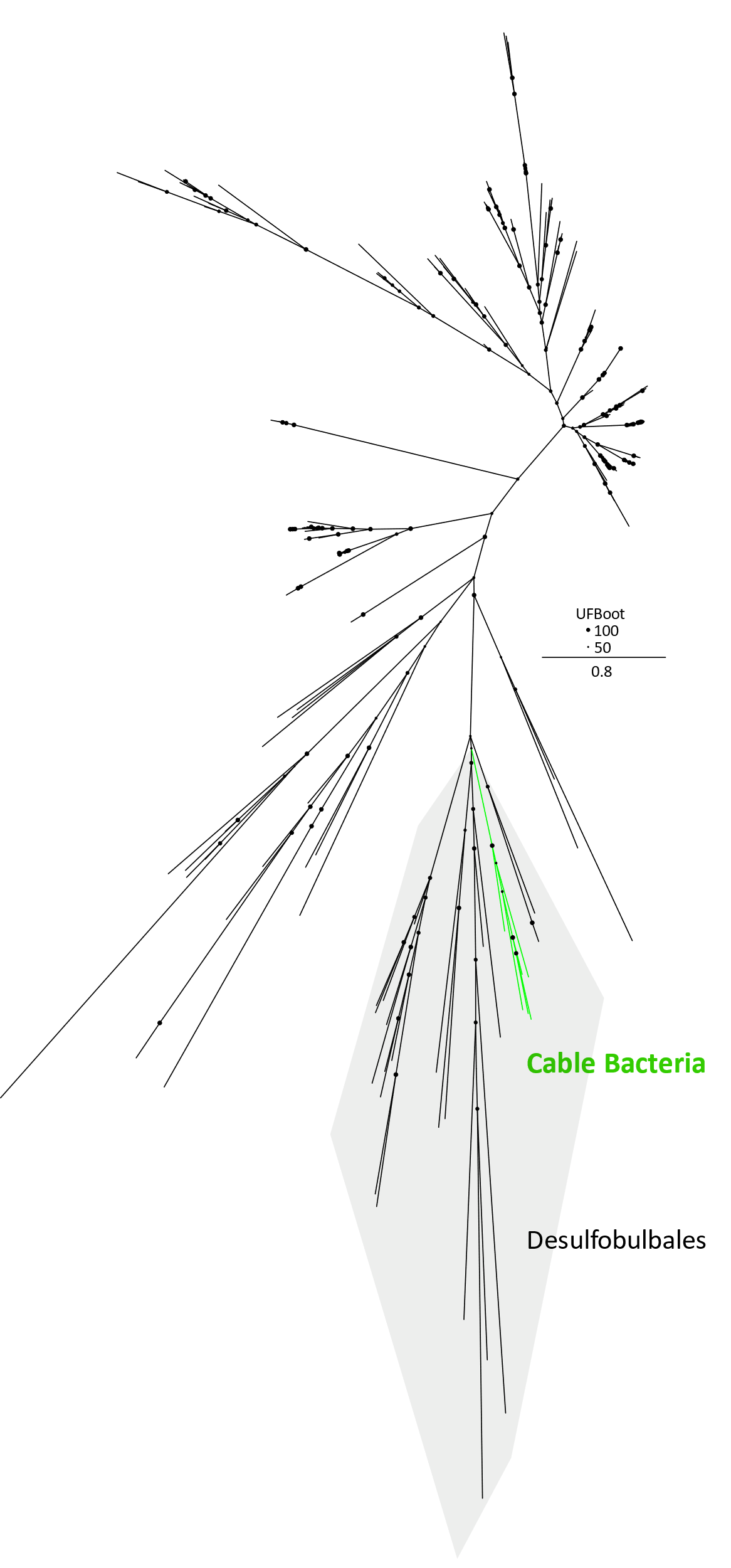


**Figure S14: Maximum likelihood phylogeny of cable bacteria and other *Desulfobulbales* CooJ protein sequences and RefSeq similarity search hits.** Phylogeny inferred using IQtree (Nguyen et al., 2015) according to the best fit model (model cpREV+F+R6). Green branches correspond to cable bacteria sequences CooJ. The grey cluster exclusively contains sequences of the Desulfobulbales order. Black circles indicate ultrafast bootstrap values.

**References**

Boschker, H. T. S., Cook, P. L. M., Polerecky, L., Eachambadi, R. T., Lozano, H., Hidalgo-Martinez, S., Khalenkow, D., Spampinato, V., Claes, N., Kundu, P., Wang, D., Bals, S., Sand, K. K., Cavezza, F., Hauffman, T., Bjerg, J. T., Skirtach, A. G., Kochan, K., McKee, M., … Meysman, F. J. R. (2021). Efficient long-range conduction in cable bacteria through nickel protein wires. *Nature Communications*, *12*(1). https://doi.org/10.1038/s41467-021-24312-4

Cornelissen, R., Bøggild, A., Thiruvallur Eachambadi, R., Koning, R. I., Kremer, A., Hidalgo-Martinez, S., Zetsche, E. M., Damgaard, L. R., Bonné, R., Drijkoningen, J., Geelhoed, J. S., Boesen, T., Boschker, H. T. S., Valcke, R., Nielsen, L. P., D’Haen, J., Manca, J. V., & Meysman, F. J. R. (2018). The Cell Envelope Structure of Cable Bacteria. *Frontiers in Microbiology*, *9*. https://doi.org/10.3389/fmicb.2018.03044

De Coster, W., D’Hert, S., Schultz, D. T., Cruts, M., & Van Broeckhoven, C. (2018). NanoPack: Visualizing and processing long-read sequencing data. *Bioinformatics*, *34*(15), 2666–2669. https://doi.org/10.1093/bioinformatics/bty149

Kaluarachchi, H., Sutherland, D. E. K., Young, A., Pickering, I. J., Stillman, M. J., & Zamble, D. B. (2009). The Ni(II)-Binding Properties of the Metallochaperone SlyD. *Journal of the American Chemical Society*, *131*(51), 18489–18500. https://doi.org/10.1021/ja9081765

Lacasse, M. J., & Zamble, D. B. (2016). [NiFe]-Hydrogenase Maturation. *Biochemistry*, *55*(12), 1689–1701. https://doi.org/10.1021/acs.biochem.5b01328

Leach, M. R., Sandal, S., Sun, H., & Zamble, D. B. (2005). Metal Binding Activity of the *Escherichia coli* Hydrogenase Maturation Factor HypB. *Biochemistry*, *44*(36), 12229–12238. https://doi.org/10.1021/bi050993j

Loy, A., Lehner, A., Lee, N., Adamczyk, J., Meier, H., Ernst, J., Schleifer, K. H., & Wagner, M. (2002). Oligonucleotide microarray for 16S rRNA gene-based detection of all recognized lineages of sulfate-reducing prokaryotes in the environment. *Applied and Environmental Microbiology*, *68*(10), 5064–5081. https://doi.org/10.1128/AEM.68.10.5064-5081.2002

Lücker, S., Steger, D., Kjeldsen, K. U., MacGregor, B. J., Wagner, M., & Loy, A. (2007). Improved 16S rRNA-targeted probe set for analysis of sulfate-reducing bacteria by fluorescence in situ hybridization. *Journal of Microbiological Methods*, *69*(3), 523–528. https://doi.org/10.1016/j.mimet.2007.02.009

Nguyen, L. T., Schmidt, H. A., Von Haeseler, A., & Minh, B. Q. (2015). IQ-TREE: A fast and effective stochastic algorithm for estimating maximum-likelihood phylogenies. *Molecular Biology and Evolution*, *32*(1), 268–274. https://doi.org/10.1093/molbev/msu300

Pfeffer, C., Larsen, S., Song, J., Dong, M., Besenbacher, F., Meyer, R. L., Kjeldsen, K. U., Schreiber, L., Gorby, Y. A., El-Naggar, M. Y., Leung, K. M., Schramm, A., Risgaard-Petersen, N., & Nielsen, L. P. (2012). Filamentous bacteria transport electrons over centimetre distances. *Nature*, *491*(7423), 218–221. https://doi.org/10.1038/nature11586

Smets, B., Boschker, H. T. S., Wetherington, M. T., Lelong, G., Hidalgo-Martinez, S., Polerecky, L., Nuyts, G., De Wael, K., & Meysman, F. J. R. (2024). Multi-wavelength Raman microscopy of nickel-based electron transport in cable bacteria. *Frontiers in Microbiology*, *15*. https://doi.org/10.3389/fmicb.2024.1208033

Wülfing, C., & Plückthun, A. (1994). Protein folding in the periplasm of *Escherichia coli*. *Molecular Microbiology*, *12*(5), 685–692. https://doi.org/10.1111/j.1365-2958.1994.tb01056.x
